# densityCut: an efficient and versatile topological approach for automatic clustering of biological data

**DOI:** 10.1093/bioinformatics/btw227

**Published:** 2016-04-23

**Authors:** Jiarui Ding, Sohrab Shah, Anne Condon

**Affiliations:** ^1^Department of Computer Science, University of British Columbia, Vancouver, BC V6T 1Z4, Canada; ^2^Department of Molecular Oncology, BC Cancer Research Centre, Vancouver, BC V5Z 1L3, Canada

## Abstract

**Motivation**: Many biological data processing problems can be formalized as clustering problems to partition data points into sensible and biologically interpretable groups.

**Results**: This article introduces densityCut, a novel density-based clustering algorithm, which is both time- and space-efficient and proceeds as follows: densityCut first roughly estimates the densities of data points from a *K*-nearest neighbour graph and then refines the densities via a random walk. A cluster consists of points falling into the basin of attraction of an estimated mode of the underlining density function. A post-processing step merges clusters and generates a hierarchical cluster tree. The number of clusters is selected from the most stable clustering in the hierarchical cluster tree. Experimental results on ten synthetic benchmark datasets and two microarray gene expression datasets demonstrate that densityCut performs better than state-of-the-art algorithms for clustering biological datasets. For applications, we focus on the recent cancer mutation clustering and single cell data analyses, namely to cluster variant allele frequencies of somatic mutations to reveal clonal architectures of individual tumours, to cluster single-cell gene expression data to uncover cell population compositions, and to cluster single-cell mass cytometry data to detect communities of cells of the same functional states or types. densityCut performs better than competing algorithms and is scalable to large datasets.

**Availability and Implementation**: Data and the densityCut R package is available from https://bitbucket.org/jerry00/densitycut_dev.

**Contact**: condon@cs.ubc.ca or sshah@bccrc.ca or jiaruid@cs.ubc.ca

**Supplementary information:** Supplementary data are available at *Bioinformatics* online.

## 1 Introduction

Clustering analysis (unsupervised machine learning), which organizes data points into sensible and meaningful groups, has been increasingly used in the analysis of high-throughput biological datasets. For example, The Cancer Genome Atlas project has generated multiple omics data for individual patients. One can cluster the omics data of individuals into subgroups of potential clinical relevance. To study clonal evolution in individual cancer patients, we can cluster variant allele frequencies of somatic mutations, such that mutations in the same cluster are accumulated during a specific stage of clonal expansion. Emerging technologies such as single-cell sequencing have made it possible to cluster single-cell gene expression data to detect rare cell populations, or to reveal lineage relationships (Pollen *et al.*, 2014; Xu *et al.*, 2015). One can cluster single-cell mass cytometry data to study intratumour heterogeneity (Levine *et al.*, 2015). As measurement technology advances have drastically enhanced our abilities to generate various high-throughput datasets, there is a great need to develop efficient and robust clustering algorithms to analyze large *N* (number of data points), large *D* (dimensions of data) datasets, with the ability to detect arbitrary shape clusters and automatically determine the number of clusters.

The difficulties of clustering analysis lie in part with the definition of a cluster. Of the numerous proposed clustering algorithms, the density-based clustering algorithms (Fraley *et al.*, 2007; Fukunaga *et al.*, 1975) are appealing because of the probabilistic interpretation of a cluster generated by these algorithms. Let $D={\left\{x_{i}\right\}}_{i=1}^{N},x_{i}\in \;R^{D}$ be drawn from an unknown density function $f(x),x\in X\subset \;R^{D}$. For model-based approaches such as Gaussian mixture models $f(x)=\sum_{c=1}^{C}\pi_{c}N(x|\mu_{c},\Sigma_{c})$, a cluster is considered as the points generated from a mixture component, and the clustering problem is to estimate the parameters of the density function from D (Fraley *et al.*, 2007). To analyze datasets consisting of complex shape clusters, nonparametric methods such as kernel density estimation can be used to estimate $\hat{f}(x)=\sum_{i=1}^{N}K_{h}(x,x_{i})$, where $K_{h}(\cdot )$ is the kernel function with bandwidth *h*. Here, a cluster is defined as the data points associated with a ‘mode’ of the density function f(x) (Wishart, 1969). The widely used ‘mean-shift’ algorithm (Cheng, 1995; Comaniciu *et al.*, 2002; Fukunaga *et al.*, 1975) belongs to this category, and it locates the modes of the kernel density function $\hat{f}(x)$ by iteratively moving a point along the density gradient until convergence. This algorithm, however, is computationally expensive, having time complexity $O(N^{2}T)$, where *T* is the number of iterations, typically dozens of iterations are sufficient for most cases. A more efficient, non-iterative graph-based approach (Koontz *et al.*, 1976) constructs trees such that each data point $x_{i}$ represents a node of a tree, the parent of node $x_{i}$ is a point $x_{j}$ which is in the direction closest to the gradient direction $\nabla \hat{f}(x_{i})$, and the root of a tree corresponds to a mode of $\hat{f}(x)$. Then each tree constitutes a cluster. This algorithm has been used to reduce the time complexity of the mean-shift algorithm to $O(N^{2})$ (Vedaldi *et al.*, 2008), and has been extended in several ways, e.g. constructing trees after filtering out noisy modes (Rodriguez *et al.*, 2014).

Nonparametric clustering methods have been generalized to produce a hierarchical cluster tree (Hartigan, 1975). Consider the λ level set of a density function *f*(*x*):

L(λ;f(x))={x|f(x)≥λ}.

The ‘high level clusters’ at level λ are the connected components of L(λ;f(x)) (in the topological sense, the maximal connected subsets of L(λ;f(x))). As λ goes from 0 to max⁡f(x), the high level clusters at all levels constitute the level set tree, where the leaves of the tree correspond to the modes of f(x) (Stuetzle *et al.*, 2010). The widely used DBSCAN algorithm (Ester *et al.*, 1996) extracts the high level clusters at just one given level λ. Many original approaches for level set tree construction in statistics (Menardi *et al.*, 2013) take the straightforward ‘plug-in’ approach to estimating the level set tree from $\hat{f}(x)$ by partitioning the feature space, i.e. X. Therefore, they are computationally demanding, especially for high-dimensional data. Recently, efficient algorithms have been proposed to partition the samples D directly (Chaudhuri *et al.*, 2010; Kpotufe *et al.*, 2011). Recovering the level set tree from a finite dataset is more difficult than partitioning the dataset into separate clusters. Correspondingly, theoretical analyses show that for these algorithms to identify salient clusters from finite samples, the number of data points *N* needs to grow exponentially in the dimension *D* (Chaudhuri *et al.*, 2010; Kpotufe *et al.*, 2011). Moreover, although the level set tree provides a more informative description of the structure of the data, many applications still need the cluster membership of each data point, which is not available directly from the level set tree.

The spectral clustering algorithm (Ng *et al.*, 2002; Shi *et al.*, 2000) works on an *N* by *N* pairwise data similarity matrix **S**, where each element $S_{i,j}$ measures the similarity between $x_{i}$ and $x_{j}$. The similarity matrix can be considered as the adjacency matrix of a weighted graph G=(V,E), where vertex *v_i_* represents $x_{i}$ and the edge weight $E_{i,j}=S_{i,j}$. Given the number of clusters *C*, the spectral clustering algorithm partitions the graph G into *C* disjoint, approximately equal size clusters, such that the points in the same cluster are ‘similar’, while points in different clusters are ‘dissimilar’. In contrast to density-based methods, the spectral clustering algorithm does not make assumptions on the probabilistic model which generates data D (Von Luxburg, 2007). Therefore, selecting the number of clusters is a challenging problem for spectral clustering algorithms, especially in the presence of outliers or when the number of clusters is large. In addition, the spectral clustering algorithm is time-consuming because it needs to compute the eigenvalues and eigenvectors of the row-normalized similarity matrix **S**, requiring $\Theta (N^{3})$ time. Instead of using single value decomposition to calculate the eigenvalues and eigenvectors, the power iteration clustering algorithm (PIC) (Lin *et al.*, 2010) iteratively smoothes a random initial vector by the row-normalized similarity matrix, such that the points in the same cluster will be similar in value. Then the *k*-means algorithm is used to partition the smoothed vector into *C* clusters. Although PIC has a time complexity of $O(N^{2}T)$, where *T* is the number of iterations, PIC may encounter many difficulties in practice. First, the points from two quite distinct clusters may have very similar ‘smoothed’ densities, and therefore they may not be distinguishable by *k*-means. Second, the points in a non-convex shape cluster can break into several clusters. As the number of clusters increases, these problems become more severe (Lin *et al.*, 2010).

In this article, we introduce a simple and efficient clustering algorithm, densityCut, which shares some advantages of both density-based clustering algorithms and spectral clustering algorithms. As for spectral clustering algorithms, densityCut works on a similarity matrix; thus it is computationally efficient, even for high-dimensional data. Using a sparse *K*-nearest neighbour graph further reduced the time complexity. Besides, we can use a random walk on the *K*-nearest neighbour graph to estimate densities at each point. As for many density-based clustering algorithms, densityCut is simple, efficient, and there is no need to specify the number of clusters as an input. Moreover, densityCut inherits both methods’ advantage of detecting arbitrarily shaped clusters. Finally, densityCut offers a novel way to build a hierarchical cluster tree and to select the most stable clustering. We first benchmark densityCut against widely used ten simulation datasets and two microarray gene expression datasets to demonstrate its robustness. We then use densityCut to cluster variant allele frequencies of somatic mutations to infer clonal architectures in tumours, to cluster single-cell gene expression data to uncover cell population compositions, and to cluster single-cell mass cytometry data to detect communities of cells of the same functional states or types.

## 2 Methods

**Fig. 1.**
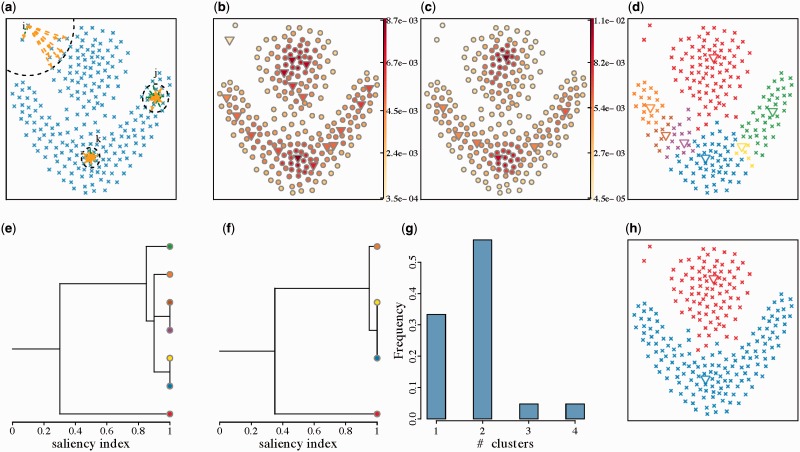
Major steps of the densityCut algorithm. (**a**) Data points in D. (This dataset was introduced by Fu *et al.*, 2007.) The dotted black circles represent the balls containing *K* = 8 points from D centred at three example points, *i*, *j* and *k*, whose densities to be estimated. Each point connects to its *K* = 8 nearest-vertices by orange arrows. Other points connect to *i*, *j* and *k* by green arrows if *i*, *j* and *k* are among the *K* in-vertices of these points. Notice the asymmetry of in-vertices and out-vertices of a vertex in a *K*nn graph, e.g. vertex *v_i_* has one in-vertex but *K* = 8 out-vertices. (**b**) Colour coded *K*nn estimated densities at points from D. The modes of densities are represented by triangles ‘▽’. (**c**) The refined densities based on a random walk. (**d**) Initial clustering by assigning data points to modes. (**e**) The tree created by merging clusters without adjusting valley heights. (**f**) The tree created by merging clusters based on the saliency index using the adjusted valley heights. (**g**) The cluster number frequency plot. (**h**) The final clustering results

The densityCut method consists of four major steps (Supplementary Algorithm 1): (i) density estimation: given a set of data points $D={\left\{x_{i}\right\}}_{i=1}^{N},x_{i}\in {\mathbb{R}}^{D}$, form a directed unweighted *K*-nearest neighbour graph and estimate the densities of data points by using the *K*-nearest neighbour density estimator; (ii) density refinement: refine the initial densities via a random walk on the unweighted *K*-nearest neighbour graph; (iii) local-maxima based clustering: detect local maxima of the estimated densities, and assign the remaining points to the local maxima; (iv) hierarchical stable clustering: refine the initial clustering by merging neighbour clusters. This cluster merging step produces a hierarchical clustering tree, and the final clustering is obtained by choosing the most stable clustering as the threshold in merging clusters varies. Figure 1 demonstrates how densityCut works on a toy example (Fu *et al.*, 2007). Below we discuss each step in detail.

### 2.1 Density estimation

We adopt the *K*-nearest neighbour density estimator to estimate the density at $x\in R^{D}$ (Fig. 1(a)):

(1) $$f_{K}(x)=\frac{(K-1)/N}{V_{K}(x)}$$

where $V_{K}(x)=V_{D}\times {\left(r_{K}(x)\right)}^{D}$ is the volume of the smallest ball centred at **x** containing *K* points from D. *V_D_* is the volume of the unit ball in the *D*-dimensional space, and $r_{K}(x)$ is the distance from **x** to its *K*th nearest neighbour. Compared to the widely used kernel density estimator, *K*-nearest neighbour density estimates are easier to compute, and also the parameter *K* is more intuitive to set than the kernel bandwidths for kernel density estimators. Here for simplicity, we only calculate the densities at data points from D, represented by $f^{0}={\left(f_{1}^{0},\ldots ,f_{N}^{0}\right)}^{T}$, where $f_{i}^{0}=f_{K}(x_{i})$ and the superscript ‘0’ indicates that this is the initial *K*nn density estimate since we will refine this density in the next section. Figure 1(b) shows the estimated densities at each data point.

When computing the *K*nn densities, we can get the *K*-nearest neighbour graph G as a byproduct with the following adjacency matrix:

(2) $$W_{i,j}=\;\{\begin{array}{ll} 1 & x_{j}\in K\text{nn}(x_{i})\\ 0 & \text{otherwise} \end{array}$$

As $x_{i}$ maps to node *v_i_* in the *K*nn graph G, we next use ‘points’ and ‘nodes’ interchangeably.

The *K*nn graph G is a directed unweighted graph. While the sets of in-vertices and out-vertices of many nodes may overlap significantly, some outliers may have few, if any in-vertices. In addition, points at the boundary of density changes may also have quite different sets of in-vertices and out-vertices because their in-vertices commonly consist of points from low-density regions while out-vertices are usually from high-density regions.

### 2.2 A random walk based density refinement

As *K*nn density estimates are based on order statistics and tend to be noisy, we next introduce a way to refine the initial *K*nn density vector $f^{0}={\left(f_{1}^{0},\ldots ,f_{N}^{0}\right)}^{T}$. Our refinement is based on the intuition that (i) a high-density vertex belongs to one of the *K*-nearest neighbours of many vertices, and (ii) a vertex tends to have high density if its in-vertices also have high densities. For example, point *k* in Figure 1(a) has nine in-vertices, and these vertices are in high-density regions, and indeed, the density of *k* is 0.0087 which is higher than average for this example. Based on the above assumptions, we get the following recursive definitions of densities:

(3) $$f_{j}^{t+1}=\alpha \sum_{i}P_{i,j}f_{i}^{t}+(1-\alpha )f_{j}^{0}$$

where α∈[0,1] specifies the relative importance of information from *v_j_*’s in-vertices and the initial density estimate $f_{j}^{0}$, which is the *K*-nearest neighbour density estimate. If each row of **P** sums to one, **P** is a Markov transition matrix, which contains the transition probability from a vertex to its *K* out-vertices. Therefore, Equation 3 defines a random walk with restart. The refined density vector is the stationary distribution of the random walk on the *K*nn graph with adjacency matrix **P**. We can row-normalize matrix **W** to get $P=D^{-1}W$, where $D=\text{diag}(\sum_{j}W_{1,j},\ldots ,\sum_{j}W_{N,j})$. Compared to the original *K*nn densities in Figure 1(b), the refined densities in Figure 1(c) have fewer ‘local maxima’ (shown as triangle points, see next section for details). Similar methods have been used in information retrieval and semi-supervised learning applications (Page *et al.*, 1999).

Equation 3 can be solved exactly in the limit by $f=(1-\alpha )f^{0}{\left(I-\alpha P\right)}^{-1}$ if α<1. Therefore, the above iterations guarantee convergence. When α = 1, $f^{t}$ converges to a left eigenvector of **P** with the maximum eigenvalue of 1. In our computational experiments, when α<1, e.g. α=0.90, Equation 3 typically converges within a few dozen iterations, and can be much faster than the case when α = 1. This rough density estimation process is faster than methods which attempt to solve density estimation to a high degree of precision since density estimation is well known to be a difficult problem. Moreover, our method can be applied to data that are presented in the form of a graph, rather than as data points over the reals.

### 2.3 Local-maxima based clustering

After obtaining densities for points, we estimate the ‘modes’ of the underlying probability density function. The modes are the ‘local maxima’ of the density function with zero gradients. For finite samples, the modes are rarely located exactly at points $x_{i}\in D$, so we use the points close to the modes instead. Mathematically, modes can be approximated by points $\left\{x_{i}|\max_{|x_{j}-x_{i}|<\epsilon }f_{j}\leq f_{i}\right\}$, where ϵ is a small distance threshold. The distance ϵ is dataset dependent and difficult to choose in practice. Instead, we can define a mode as a vertex whose density is the largest among all of its in-vertices:

(4) $$\left\{v_{j}|\forall P_{i,j}>0,f_{i}<f_{j}\right\}$$

Here, we use in-vertices instead of out-vertices in order to be able to detect small cluster with less than *K* data points. As the vertices of a small cluster with less than *K* vertices can form a clique (or are highly connected to each other) in the *K*nn graph, we can detect this small cluster based on the definition of local maxima above if these points are not among the *K*-nearest neighbours of points outside this cluster. A potential problem is that some outliers with very few in-vertices could be detected as local maxima. We simply remove those local maxima whose numbers of in-vertices are less than K/2. In other words, we treat a cluster less than K/2 in size as an ‘outlier’ cluster, and densityCut is unlikely to detect this small cluster.

Data points that fall into the basin of attraction of each mode constitute a cluster. This process can be done by moving each point along its gradient direction to reach a mode. We modify the efficient graph-based hill-climbing algorithm (Koontz *et al.*, 1976) to build a unique forest (a set of trees) for a given dataset. The parent of vertex *v_i_* is defined as

(5) $$\text{Parent}(v_{i})=\arg\underset{v_{j}\in N_{i}}{\min}(|d_{j}-d_{i}||f_{i}<f_{j})$$

where $N_{i}$ is the set of in-vertices of *v_i_*. In other words, the parent of *v_i_* is the vertex which is closest to *v_i_* among all of *v_i_*’s in-vertices that have higher densities than *v_i_*. From the construction of the trees, we can see that each vertex is associated with just one tree. Therefore, each tree can be considered as a cluster. Figure 1(d) shows the clusters by assigning data points to the seven local maxima.

### 2.4 Hierarchical stable clustering

We then generate a hierarchical cluster tree and select the most stable clustering. First the density of the root of a tree T generated above is called the height of this tree, denoted by $h_{T}$, which has the largest density among all the vertices in T. Then we define the boundary points between trees $T_{1}$ and $T_{2}$:

(6) $$B(T_{1},T_{2})=\{v\in T_{1}|\exists u\in N_{v}\cap T_{2},f_{v}<f_{u}\}$$

Sets $B(T_{1},T_{2})$ and $B(T_{2},T_{1})$ are not the same: $B(T_{1},T_{2})\subset T_{1}$ and $B(T_{2},T_{1})\subset T_{2}$. The valley separating two trees is:

(7) $$\text{Valley}(T_{1},T_{2})=B(T_{1},T_{2})\cup B(T_{2},T_{1}),$$

The height of the valley separating $T_{1}$ and $T_{2}$ is defined as

(8) $$h_{\text{Valley}(T_{1},T_{2})}=\underset{v\in \text{Valley}(T_{1},T_{2})}{\max}f_{v}$$

The saliency index *ν* of a valley represents the relative height of the valley compared to the shorter tree:

(9) $$\nu (T_{1},T_{2})=\frac{h_{\text{Valley}(T_{1},T_{2})}}{\min(h_{T_{1}},h_{T_{2}})}$$

**Fig. 2.**
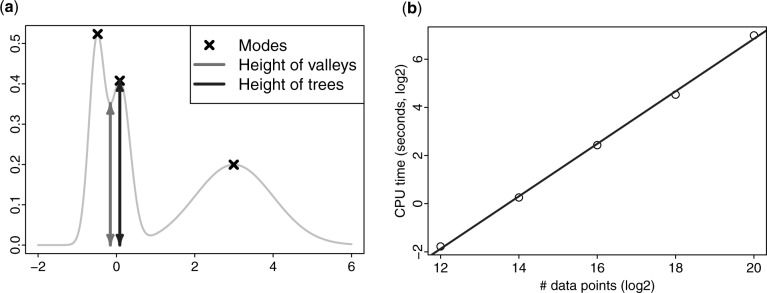
(**a**) Merging the first two trees (clusters) based on the relative height of the valley separating the two trees. (**b**) The time (in seconds, log_2_ transformed) increases almost linearly with the number of data points (log_2_ transformed)

Figure 2(a) shows the height of the valley (the length of the grey arrow) separating two adjacent trees, and the saliency index is the ratio between the length of the grey arrow and the black arrow.

The saliency index defined in Equation 9 has several properties. First, 0≤ν≤1, and *ν* is invariant under scaling of the densities by a positive constant factor. This ‘scale-free’ property is very useful for us to select a threshold for merging trees. Second, it automatically scales to the local densities of trees, e.g. to get the same saliency index, the height of the valley separating two trees in low-density regions is shorter than that separating two trees in high-density regions.

We can merge two clusters if the saliency index between them is above a threshold *ν*. When the saliency index *ν* = 1, no clusters get merged, and when *ν* = 0, all the connected clusters get merged to form a single cluster. Therefore, by gradually decreasing the saliency index threshold, we can get a hierarchical clustering tree, which is useful for us to interpret the structure of data, especially for high-dimensional data. Figure 1(e) shows the cluster tree from merging neighbouring clusters in Figure 1(d).

For a dataset consisting of clusters whose densities are considerably different, a single *K* for density estimation may be insufficient. This is because the points from a high-density cluster need a larger *K* to estimate their densities, compared to the points from a low-density cluster. The high-density cluster could ‘break’ into several small clusters when *K* is small. Instead of picking the parameter *K* on a data point basis, we can adjust the height of a valley by:

(10) $${\hat{h}}_{\text{Valley}(T_{1},T_{2})}=(1+\sum_{i}\frac{f_{i}<h_{\text{Valley}(T_{1},T_{2})}}{N})h_{\text{Valley}(T_{1},T_{2})}$$

The intuition behind this density adjustment step is that a high-density valley could be an artefact caused by splitting a high-density cluster. Figure 1(f) shows the cluster tree using the saliency indexes based on the adjusted valley heights. A potential problem of this step is that it also increases the valley height between two genuine clusters and increases their likelihood to merge. For example, as can be seen from Figure 1(e, f), the two clusters merge at saliency index around 0.30 before valley adjustment, but merge at saliency index around 0.35 after valley adjustment. We will further investigate the influence of this density adjustment step on clustering in later sections.

We finally introduce a method to determine the number of clusters to produce the final clustering from the hierarchical cluster tree. The basic idea is that by gradually decreasing the saliency index, clusters will get merged. Noisy, non-salient clusters will get merged quickly, and true clusters will exist for a long period of time. We therefore can calculate the length of saliency index change for producing a fixed number of clusters, and select the cluster number which spans the longest saliency index changes. In our current implementation of densityCut, we decrease the saliency index evenly and therefore we can interpret the saliency index change interval as ‘Frequency’. Figure 1(g) shows the cluster number frequency plot, and Figure 1(h) shows the final clustering by merging the initial clustering to produce two clusters as selected by the cluster number frequency plot.

### 2.5 Complexity analysis and implementation

densityCut has been implemented in the statistical computation language R. densityCut has a worst-case time complexity of $O(NK+C^{2})$ and a space complexity of $O(NK+C^{2})$, where *N* is the number of data points, *K* is the number of neighbours and *C* is the number of clusters (local maxima). In practice, as a majority of clusters are only adjacent to few clusters, the time and space complexity is typically of O(NK+C). We did not consider the time used to compute the *K*nn graph in densityCut as numerous algorithms have been developed for efficient *K*nn search with different complexities, and typically it takes less time to compute the *K*nn graph compared to cluster the data. To build the *K*nn graph given a data matrix, efficient algorithms such as kd-trees can be used in low-dimensional spaces (D≤20) with time complexity O(Nlog⁡(N)) (Mount, 1998). To build the *K*nn graph in high-dimensional spaces (*D* < 1000), efficient software libraries based on random projection exist to repeatedly partition the data to build a tree (https://github.com/spotify/annoy). This algorithm can run in *O*(*NDT*) time, where *T* is the number of trees, and typically dozens of trees are enough to preserve the accuracy of *K*nn search.

To demonstrate the scalability of densityCut, we tested densityCut on a Mac desktop computer running OS X Version 10.9.5. The computer has 32 GB of RAM and a 3.5 GHz four-core Intel i7 processor with 8 MB cache. We carried out all the experiments presented in the article on this computer.

We sampled $\left\{2^{12},2^{14},2^{16},2^{18},2^{20}\right\}$ data points from a mixture of 64 two-dimensional Gaussian distributions. As can be seen from Figure 2(b), the running time increased almost linearly in the number of data points. It took about 127 CPU seconds to cluster a million data points ($N=2^{20}$).

### 2.6 Parameter setting

Our algorithm has two parameters: the number of nearest neighbours *K*, and the damping factor *α* in density refinement. *K* should be small enough to detect local maxima, e.g. smaller than the number of data points in a cluster. However, very small *K* can result in poor density estimates and produce large numbers of clusters, thus ‘overfitting’ the data, and there may not exist a ‘gap’ in the cluster number frequency plot for us to select the number of clusters. On the contrary, for large *K*, densityCut may fail to detect detailed structures thus ‘underfitting’ the data.

Theoretical analysis for spectral clustering shows that *K* should be Ω(log⁡(N)) to produce a connected graph (Von Luxburg, 2007), and limit results are obtained under conditions K/log⁡(N)→∞ and K/N→0. *K* is also dependent on the dimensionality *D*. For the density estimate at **x** (f(x) is Lipchitz smooth in a neighbour of **x**) from its *K*-nearest neighbours, under conditions $k/N^{2/(2+D)}\to 0$ and k→∞, we can get $|\hat{f}(x)-f(x)|≲f(x)/\sqrt{k}$ (Dasgupta *et al.*, 2014).

In practice, *K* should be dataset dependent. For example, if the Euclidean distance is used, *K* should be sufficiently small such that the Euclidean distance is a good measure of the distance between two close data points even the data lie in a manifold. If the number of clusters is small, *K* should increase to prevent generating too many local maxima. We therefore conducted an empirical study of the influence of *K* and *α* on clustering the data in Figure 1, for which *N* = 240 (Supplementary Figs S1 and S2). First, when $K=\log_{2}(N)=8$, densityCut correctly detected the two clusters given different values for *α* (Supplementary Fig. S1). Small $K=\log_{2}(N)=4$ produced ‘spiky’ density estimates and resulted in many local maxima (Supplementary Fig. S1). On the contrary, large *K* produced flat density estimates, and the two true clusters tended to merge because of no deep valley between them (Supplementary Fig. S1). We therefore used a default value of $K=\log_{2}(N)$. In addition, when α=0.9 or 0.99, densityCut correctly detected the two clusters given different values for *K*. Increasing α produced better clustering results but it took much longer for the density refinement step to converge, e.g. median 176 iterations when α=0.99 compared to 41 iterations when α=0.90. We set the default value for α=0.90 as it made a good compromise between accuracy and execution time.

The valley height adjustment step plays a role of ‘smoothing’ the density estimates. This functionality is especially useful for small *K*. For example, even when $K=0.5\log_{2}(N)=4$, densityCut correctly detected the two clusters after adjusting the heights of valleys (Supplementary Fig. S2). For all the results presented in this article, we used the default parameter setting ($K=\log_{2}(N)$ and α=0.9) with the valley height adjustment step.

## 3 Results

### 3.1 Benchmarking against state-of-the-art algorithms

#### 3.1.1 Synthetic datasets

We benchmarked densityCut against state-of-the-art clustering algorithms for biological datasets (Wiwie *et al.*, 2015) on synthetic datasets that have been widely used to evaluate clustering algorithms. Specifically, we compared densityCut with three best algorithms reported in Wiwie *et al.* (2015) (Supplementary Materials), i.e. the hierarchical clustering algorithm (HC, from the R stats package) with average linkage, the partitioning around medoids (PAM, from the R cluster package) algorithm and the density-based clustering algorithm OPTICS (from the R dbscan package). We also compare densityCut with the Gaussian mixture model (GMM, from the R mclust package) based clustering algorithm and the normalized cut (NCut, from the R kernlab package) spectral clustering algorithm. We used ten synthetic datasets (the eight ‘shape’ datasets and the two most challenging ‘S-sets’), downloaded from http://cs.joensuu.fi/sipu/datasets/. Seven out of the ten datasets were also used in Wiwie *et al.* (2015) (except for the most challenging case ‘S4’ from the S-set, the challenging ‘Jain’ dataset and the ‘D31’ dataset with large number of clusters – 31 clusters). For the algorithms requiring the number of clusters as a input parameter (PAM, GMM and NCut), we set the number of clusters as the ground truth cluster number for each dataset. For HC, we cut the dendrogram to produce the ground truth number of clusters for each dataset.

To measure clustering performance, we compared the clustering results from each algorithm to the ground truth to compute the maximum-matching measure (MMM, range from 0 to 1), the normalized mutual information (NMI, range from 0 to 1) and the adjusted Rand index (ARI, with expected value of zero and maximum value of one). For all these measures, high values mean high similarity between two sets. More information about these measures can be found in Supplementary Materials.

**Fig. 3.**
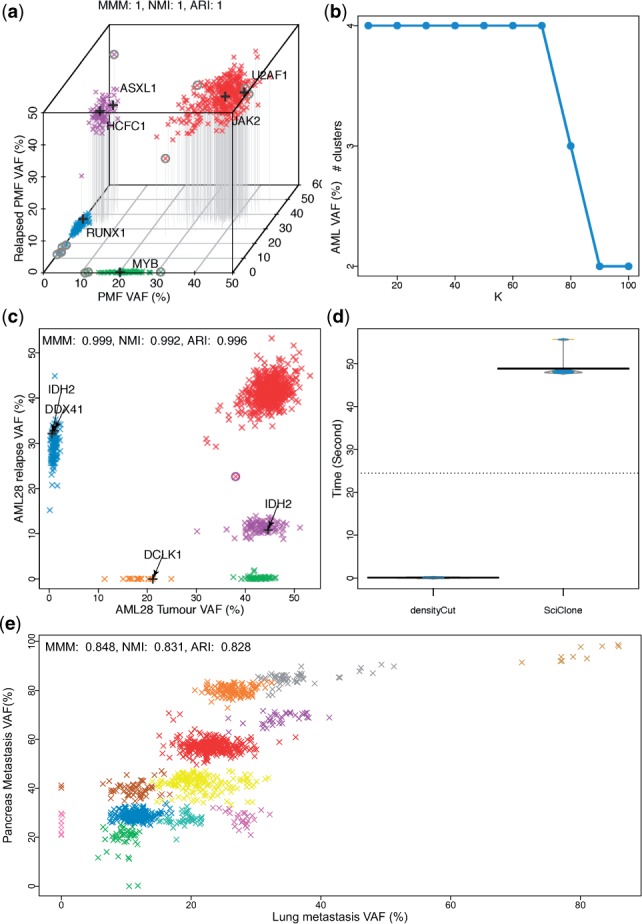
Clustering variant allele frequencies (VAF) of somatic mutations. (**a**, **b**) Clustering multi-time sample data from initial primary myelofibrosis (PMF), later acute myeloid leukaemia (AML) and after treatment relapsed PMF using densityCut. (**c**, **d**) Clustering the somatic mutations from sequencing a primary/relapse pair of an AML patient. (**e**) Clustering the somatic mutations from sequencing a lung/pancreas metastasis pair of a melanoma patient. The possible ‘driver’ mutations in each cluster are labeled with a black plus sign ‘+’. The clustering validation indices (MMM, NMI and ARI) were from comparing densityCut results with sciClone results or the results reported in the original studies. (a) Three-dimensional VAF plot. The mutations in each cluster were assigned a unique colour. The mutations with a circle ‘°’ were considered as outliers in the original publication (Engle *et al.*, 2015) before clustering analysis. (b) The number of clusters produced by densityCut as we gradually increased *K* from $\log_{2}(N)$ to $10\log_{2}(N)$. (c) The mutation assigned to the violet colour cluster by sciClone but assigned to the red colour cluster by densityCut was labeled with a circle ‘°’. (d) densityCut and sciClone execution time based on repeated ten runs. The hierarchical clustering trees and the cluster number frequency plots are in Supplementary Figure S7

As shown in Supplementary Figure S3, densityCut performed the best in terms of the above evaluation measures (with mean MMM, NMI and ARI of 0.911, 0.897 and 0.854). Overall, the clustering results on these synthetic benchmark datasets demonstrated that densityCut can produce excellent results if the high-density clusters were separated by low-density valleys. For the datasets in Supplementary Figure S3(a, c–e, g–j), densityCut detected the right number of clusters and revealed the structures of these datasets. The red colour cluster in Supplementary Figure S3(b) was considered as two separated clusters originally (Zahn, 1971). However, the sparse background points and the centre high-density points could be considered as from the same cluster for density-based clustering. For the dataset in Figure 3(f), densityCut failed to detect the three clusters because there were no ‘deep’ valleys between the outer arc cluster and the two Gaussian clusters. Therefore, the outer arc cluster got merged to the right Gaussian cluster. Without the valley height adjustment step, densityCut generated the same clustering (Supplementary Fig. S4). The density-based clustering algorithm OPTICS ranked second with mean MMM, NMI and ARI of 0.840, 0.717 and 0.685, respectively (Supplementary Fig. S3, last column; Supplementary Fig. S5(a)).

PAM and GMM performed poorly on the datasets consisting of irregular shape clusters (Supplementary Fig. S3(a–f), last column). PAM results on these datasets had mean MMM, NMI and ARI of 0.687, 0.492 and 0.414, respectively, and GMM results on these datasets had mean MMM, NMI and ARI of 0.617, 0.441 and 0.311, respectively. However, PAM did very well on the datasets where the points in each cluster were sampled from a two-dimensional Gaussian distribution with mean MMM, NMI and ARI of 0.908, 0.870 and 0.828 (Supplementary Fig. S3(g–j), last column). In contrast, GMM performed inferior to PAM on these datasets with mean MMM, NMI and ARI of 0.787, 0.826 and 0.716. For example, for ‘D31’ in Supplementary Figure S5(e), cluster two consisted of only a single data point, and cluster nine consisted of two data points. Cluster three and 18 consisted of points from multiple Gaussian distributions. One major reason for the failure of GMM was that the relatively large number of clusters (31) compared to the limited number of data points (3100) and the overlapped clusters resulted in many local maxima in its objective log-likelihood function, while the Expectation–Maximization algorithm for fitting GMM only searched for a local maximum of the objective function. By contrast, densityCut directly located the high-density peaks and selected the most stable clustering, and thus it was less likely influenced by spurious density peaks.

Both HC and NCut can cluster datasets consisting of arbitrary shape clusters. On the datasets consisting of non-convex shape clusters, HC (with mean MMM, NMI and ARI of 0.788, 0.589 and 0.577) and NCut (with mean MMM, NMI and ARI of 0.771, 0.628 and 0.55) performed slightly better than PAM and GMM (Supplementary Fig. S3(a–f), last column). On the datasets consisting of convex shape clusters, HC (with mean MMM, NMI and ARI of 0.810, 0.841 and 0.746) and NCut (with mean MMM, NMI and ARI of 0.746, 0.814 and 0.678) performed slightly worse than PAM and GMM (Supplementary Fig. S3(g–j), last column). Compared to HC and NCut, densityCut performed better in both the datasets consisting of arbitrary shape clusters (with mean MMM, NMI and ARI of 0.922, 0.919 and 0.886) and the datasets consisting of convex shape clusters (with mean MMM, NMI and ARI of 0.895, 0.863 and 0.807). Moreover, densityCut had low complexity and was scalable to large datasets.

#### 3.1.2 Microarray gene expression data

Gene expression data have been used to stratify cancer patients into biologically or clinically meaningful subtypes, e.g. different subtypes of patients have distinct prognosis. Here, we tested densityCut on the two microarray gene expression datasets as in Baek *et al.* (2011) (Supplementary Materials). The results in Supplementary Figure S6 show that densityCut performs the best on these two datasets (with MMM, NMI and ARI of 0.926, 0.780 and 0.853 on dataset one, and 0.981, 0.860 and 0.924 on dataset two) compared with PAM, HC, OPTICS, NCut and GMM.

Although OPTICS produced good results on the previous two dimensional synthetic datasets, it performed poorly on these high-dimensional gene expression datasets (ten dimensions are considered as high dimensions for density-based clustering in Kriegel *et al.*, 2011). OPTICS produced just one cluster for each dataset. Although the absolute distances between data points are not discriminative in high-dimensional spaces (the curse of dimensionality, the distances between any two points are approximately the same), the relative distances (the order of closeness) could still be meaningful, and could be captured by the *K*nn graph. densityCut explores the topology of the *K*nn graph thus performed better on high-dimensional spaces than OPTICS. GMM (with MMM, NMI and ARI of 0.626, 0.621 and 0.408 on dataset one, and 0.962, 0.765 and 0.850 on dataset two) and PAM (with MMM, NMI and ARI of 0.714, 0.583 and 0.411 on dataset one, and 0.952, 0.727 and 0.815 on dataset two) performed relatively well on these datasets. The results were consistent with the results from the previous study that GMM and PAM (more precisely, the *k*-means algorithm) performed well on clustering gene expression data (de Souto *et al.*, 2008).

### 3.2 Inferring clonal architectures of individual tumours

Cancer cells are heterogeneous, and a subpopulation of cancer cells of the same patient could harbour different sets of mutations (Miller *et al.*, 2014). Moreover, cancer cells frequently accumulate additional mutations after treatment or in metastasis. Understanding the clonal architecture of each tumour provides insights into tumour evolution and treatment responses. We used densityCut to cluster the somatic variant allele frequencies (VAF) measured from DNA sequencing of multiple tumour biopsies. The mutations in each cluster were accumulated during a specific stage of clonal expansion. The clustering results provide valuable information of the clonal architectures of tumours.

We first tested densityCut on the mutation data from a primary myelofibrosis (PMF) patient (Engle *et al.*, 2015). This patient was first diagnosed with PMF, and seven years later, this patient’s tumour transformed to acute myeloid leukaemia (AML). After chemotherapy treatment, the patient underwent complete remission. However, 1.5 years later, the patient redeveloped PMF but no evidence of AML relapse. A total of 649 single nucleotide variants detected in whole genome sequencing of either PMF, AML or relapse PMF genomes were validated by targeted high-coverage sequencing. We used densityCut to jointly cluster the targeted sequencing VAFs from PMF, AML and relapse PMF tissues. Figure 3(a) shows that densityCut grouped the mutations into four clusters. The hierarchical clustering trees and the accompanying cluster number frequency plots are in Supplementary Figure S7(a).

Overall, densityCut clustering results matched those presented in the original study. However, to produce the results, the authors (Engle *et al.*, 2015) used different algorithms and several pre-processing steps. For example, the authors used DBSCAN (Ester *et al.*, 1996) to detect outliers (the mutations with circles ‘°’ in Fig. 3(a)), and then used Mclust (Fraley *et al.*, 2007) for model selection and final clustering analysis. The maximum number of clusters was limited to four, and each cluster had to contain at least seven mutations (Engle *et al.*, 2015). In contrast, we directly used densityCut to cluster the VAFs and produced exactly the same results (MMM = 1, the outliers were not considered in calculating MMM.) We also changed the parameter *K* from the default $\log_{2}(N)=10$ to $2\log_{2}(N)$ until $10\log_{2}(N)$. Only after *K* was set to $8\log_{2}(N)$, the red colour cluster and the violet colour cluster got merged, as can be seen from Figure 3(b). For $K<8\log_{2}(N)$, densityCut produced the same four clusters. OPTICS, PAM, HC, NCut and GMM produced the same results as densityCut results (to be exact, only PAM assigned one point to different clusters, Supplementary Fig. S8(c)). However, except for OPTICS, these algorithms either need the number of clusters as input parameters or cut the dendrogram to produce the desired number of clusters (HC).

Next, we tested densityCut on the acute myeloid leukaemia sample AML28 (Ding *et al.*, 2012). We jointly clustered the VAFs from sequencing both the primary tumour and the relapse tumour after 26 months of chemotherapy (Ding *et al.*, 2012). Figure 3(c) shows that densityCut grouped the 804 detected somatic mutations into five clusters. The results matched those predicted by sciClone (Miller *et al.*, 2014), a variational Bayesian mixture model based clustering algorithm. Only one mutation (with circle ‘°’ in Fig. 3(c)) was assigned to the red cluster by densityCut, but originally assigned to the cyan cluster by sciClone (MMM = 0.999). densityCut is much more efficient than sciClone as can be seen from Figure 3(d). sciClone took a median of 48.12 s to run on the AML28 dataset while densityCut took a median of 0.074 s to run. Because it took less than a CPU second to run densityCut, we ran both algorithms ten times to get a more accurate estimation of the time used. For competing algorithms, PAM split the large cluster into two clusters because it tends to generate equal-size clusters (with MMM, NMI and ARI of 0.619, 0.509 and 0.767; Supplementary Fig. S8(g)). HC assigned some ‘outliers’ to a distinct clusters, and merged the points from two clusters (with MMM, NMI and ARI of 0.939, 0.964 and 0.929; Supplementary Fig. S8(h)). Similarly, GMM modelled the outliers using a Gaussian component (with MMM, NMI and ARI of 0.925, 0.865 and 0.891; Supplementary Fig. S8(j)). OPTICS results differed from densityCut results by the assignment of only one data point (Supplementary Fig. S8(f)), and NCut produced the same results as densityCut results (Supplementary Fig. S8(i)).

Finally, we used densityCut to cluster the somatic mutations from whole genome sequencing of the lung/pancreatic metastasis pair from the same melanoma patient MEL5 (Ding *et al.*, 2014). Compared to blood cancer genomes, melanoma genomes are much more complex, frequency harbouring copy number alterations. The combinations of copy number alterations, homozygous mutations and heterozygous mutations make it a challenging task to develop a model to uncover the clonal structure of these cancer genomes (Roth *et al.*, 2014). The densityCut clustering results in Figure 3(e) show that the mutations in MEL5 could be grouped into 12 clusters, providing the starting point for detailed inspection of the clonal structure of this cancer genome. Additional information such as copy number alterations would be required to fully interpret the clonal architectures. We also ran sciClone, which produced ten clusters (Supplementary Fig. S9). Both algorithms agreed in clustering 84.8% of the mutations (MMM: 0.848, Fig. 3(e)). densityCut clustering had an average silhouette width of 0.58 (Supplementary Fig. S10), which was higher than sciClone clustering average silhouette width of 0.55 (Supplementary Fig. S11). Other competing algorithms performed inferrer to densityCut with PAM and OPTICS performed second and third with average silhouette widths of 0.548 and 0.539, respectively (Supplementary Fig. S8(m, k)).

### 3.3 Clustering single-cell gene expression datasets

We used densityCut to cluster two single-cell mRNA gene expression datasets. The first dataset consists of the low-coverage mRNA expression of 23 730 genes in 301 cells from 11 populations (Pollen *et al.*, 2014). The second dataset consists of the single-cell mRNA expression of 43 309 genes in 223 stem cells from the subventricular zone of eight-week-old male mice (Llorens-Bobadilla *et al.*, 2015). We did several pre-processing steps to only select a subset of genes (Pollen *et al.*, 2014) for clustering analysis because the high technique noise in single-cell gene expression data (e.g. loss of cDNA in reverse transcription and bias in cDNA amplification) and *K*nn search in high dimensional spaces is still time-consuming. Specifically, we only kept the genes expressed in more than five cells because it is difficult to detect clusters less than five in size given the relatively large number of cells. Here, a gene was considered to be expressed in a cell if its reads per kilobase per million (RPKM) value (or fragments per kilobase per million (FPKM) value for dataset two (Llorens-Bobadilla *et al.*, 2015)) was greater than or equal to one in the cell. We then further normalized the RPKM values by log transformation: $\log_{2}(x+1)$. Here, *x* was the original RPKM value of a gene in a cell. A small value of one was added to prevent taking the log of zero or generating very small numbers.

**Fig. 4.**
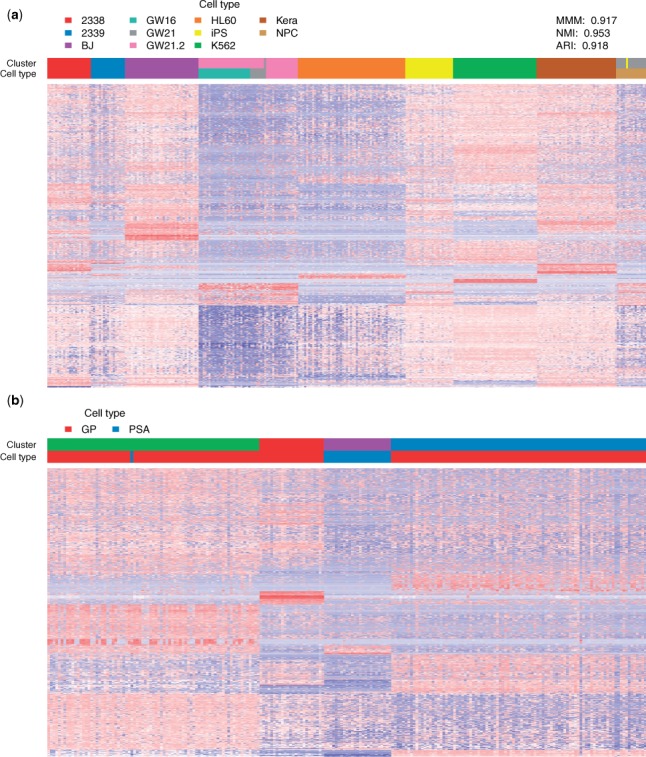
Clustering single-cell gene expression data. Each row is a gene and each column is a cell. The cell type and cluster membership of each cell are colour coded. Heatmaps show clustering (**a**) 301 cells from 11 populations and (**b**) 223 stem cells from the subventricular zone of eight-week-old male mice

Figure 4(a) shows that densityCut produced nine clusters for dataset one (MMM: 0.917, NMI: 0.953 and ARI: 0.918). densityCut cannot distinguish the cells from GW16, GW21 and GW21.2 based on the 1000 genes. These cells were quite similar as they were all from the human cortex (GW16 cells were from the germinal zone of human cortex at gestational week 16, GW21 cells were from GW21 and GW21.2 cells were cultured cells of a subset of the GW21 cells (Pollen *et al.*, 2014)). These cells could possibly be separated by selecting a better set of features for clustering analysis. In addition, one GW21 cell was misclassified as a neural progenitor cell (NPC), and one NPC was in the human-induced pluripotent stem (iPS) cell cluster. For the other seven types of cells, densityCut perfectly put them into separate clusters. Supplementary Figure S12 shows the hierarchical cluster trees and the cluster number frequency plots. Other clustering algorithms such as OPTICS, PAM, HC, NCut and GMM had inferior performance compared to densityCut results with PAM ranked second with MMM, NMI and ARI of 0.877, 0.916 and 0.854, respectively (Supplementary Fig. S13(a)). densityCut grouped the 223 stem cells of dataset two into four clusters (Fig. 4(b)). Glutamate aspartate transporter^+^/Prominin1^+ ^(GP) cells and polysialylated-neural cell adhesion molecule^+ ^(PSA) cells were in separate clusters (except for one PSA cell). The GP cells were subdivided into three clusters, consistent with the original finding that the GP cells consisted of at least three subtypes of stem cells. We next used t-SNE (Van der Maaten *et al.*, 2008) to project the 1000-dimensional single-cell gene expression data to a two-dimensional space (Supplementary Fig. S14). The results also show four very distinct clusters. Compared with the original analysis using hierarchical clustering coupled with principle component analysis feature section (Llorens-Bobadilla *et al.*, 2015), densityCut can be used in a more unbiased way to cluster single-cell gene expression data and produce the same results. On this dataset, densityCut, PAM, HC and GMM results had average silhouette widths of 0.190, 0.190, 0.191 and 0.189, respectively (Supplementary Fig. S13(b))

### 3.4 Clustering single-cell mass cytometry datasets

Finally, we used densityCut to cluster two benchmark single-cell mass cytometry (aka CyTOF) datasets (Levine *et al.*, 2015). The first dataset consists of CyTOF data of bone marrow mononuclear cells from a healthy individual. Manually gating assigned 81 747 cells to 24 cell types based on 13 measured surface protein markers (Bendall *et al.*, 2011). Dataset two contains CyTOF data from two healthy adult donors H1 and H2. For H1, manual gating assigned 72 463 cells to 14 cell types based on 32 measured surface protein markers. Manual gating assigned 31 721 cells to the same 14 cell populations from H2 based on the 32 surface protein markers. These manually identified cell populations were used as ground truth to test densityCut.

**Fig. 5.**
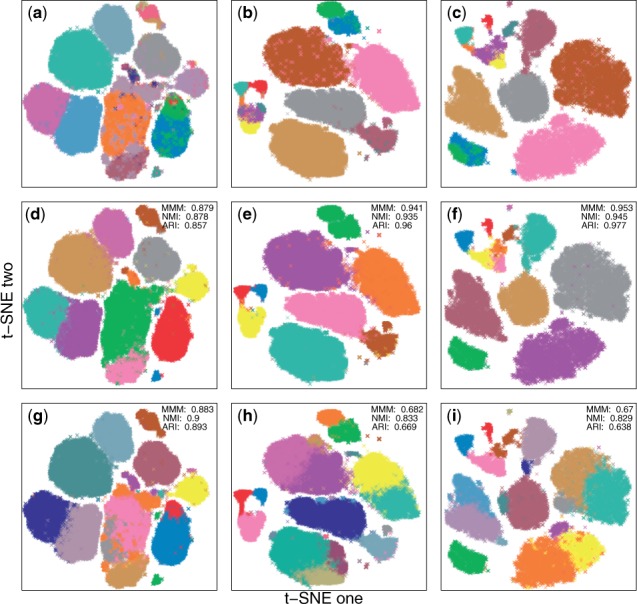
Comparing densityCut and PhenoGraph in clustering single-cell mass cytometry data. The original high-dimensional mass cytometry data were projected onto two dimensional spaces by t-SNE just for visualization purpose. Cell types and clustering memberships of data points were colour coded. (**a**–**c**) The ground truth. (**d**–**f**) densityCut clustering results. (**g**–**i**) PhenoGraph clustering results

We compared densityCut to the recently proposed algorithm, the PhenoGraph algorithm (Levine *et al.*, 2015), in clustering the benchmark single-cell CyTOF datasets. As both densityCut and PhenoGraph first build a *K*nn graph, we used the same $K=\log_{2}(N)$ for both algorithms. As can be seen from Figure 5, densityCut detected 12 distinct cell types (clusters) in dataset one, 9 cell types in H1, and 12 cell types in H2 (the hierarchical clustering trees and the cluster number frequency plots are in Supplementary Fig. S15). The PhenoGraph algorithm detected 18, 24 and 20 clusters in dataset one, H1 and H2, respectively. Based on MMM, NMI and ARI, densityCut performed slightly worse on dataset one (MMM: 0.879 versus 0.883, NMI: 0.878 versus 0.900 and ARI: 0.857 versus 0.893), but performed better on H1 (MMM: 0.941 versus 0.682, AMI: 0.935 versus 0.833 and ARI: 0.96 versus 0.669) and H2 (MMM: 0.953 versus 0.67, NMI: 0.945 versus 0.829 and ARI: 0.977 versus 0.638). As for efficiency, densityCut was around two times faster than PhenoGraph based on the current implementations (Supplementary Fig. S16). Other clustering algorithms such as PAM, HC, NCut and GMM are not scalable to these relatively large datasets. For example, we can only run OPTICS on the first dataset (OPTICS took about 17 minutes while densityCut took only 24 s).

## 4 Conclusions and discussion

We developed densityCut, a simple and efficient clustering algorithm. densityCut effectively clustered irregular shape synthetic benchmark datasets. We have successfully used densityCut to cluster variant allele frequencies of somatic mutations, single-cell gene expression data and single-cell CyTOF data. densityCut is based on density estimation on graphs. It could be considered as a variation of the spectral clustering algorithms but is much more time- and space-efficient. Moreover, it automatically selects the number of clusters and works for the datasets with a large number of clusters. In summary, densityCut does not make assumptions about the shape, size and the number of clusters, and can be broadly applicable for exploratory data analysis.

A recent study has shown that current strategies for whole genome sequencing studies missed many somatic mutations (Griffith *et al.*, 2015). By increasing the sequencing depths from 30× in their original study (Ding *et al.*, 2012) to 300× and using a consensus of somatic single nucleotide variant (SNV) callers, the number of identified SNVs increased from 118 to 1343. Based on the 1343 SNVs, they identified two extra sub-clones (Griffith *et al.*, 2015). Moreover, an additional 2500 SNVs were highly likely to be genuine somatic SNVs but still without enough evidence even at 300× coverage. For more complex genomes such as melanoma and breast cancer genomes, the number of SNVs could be much larger. Therefore, efficient algorithms such as densityCut are necessary to infer the clonal structures in individual tumours as more genomes are sequenced at higher coverage in the near future.

In recent years, single-cell techniques have empowered scientists to investigate cellular heterogeneity. Computational tools are necessary to analyze these single-cell measurements with high dimensionality and large numbers of cells. Efficient algorithms such as densityCut whose computational complexities are independent of the dimensionality of data, and can cluster millions of points in a few minutes can be valuable tools to process these datasets to distill single cell biology.

## Supplementary Material

Supplementary Data
