# Supplementary material for: densityCut: an efficient and versatile topological approach for automatic clustering of biological data
Source: Bioinformatics. 2016 Apr 23;32(17):2567–76. doi: 10.1093/bioinformatics/btw227 (PMC5013902; doi:10.1093/bioinformatics/btw227)
Supplement: Supplementary Data [file supp_32_17_2567__index.html]

densityCut: an efficient and versatile topological approach for automatic clustering of biological data — densityCut: an efficient and versatile topological approach for automatic clustering of biological data — Supplementary Data 

# densityCut: an efficient and versatile topological approach for automatic clustering of biological data

## Supplementary Data

files

- Supplementary Data - pdf file
