# Supplementary material for: densityCut: an efficient and versatile topological approach for automatic clustering of biological data
Source: Bioinformatics. 2016 Apr 23;32(17):2567–76. doi: 10.1093/bioinformatics/btw227 (PMC5013902; doi:10.1093/bioinformatics/btw227)
Supplement: Supplementary Data [file supp_btw227_supp_densitycut.pdf]

Supplementary material to "densityCut: an efficient and versatile topological approach for automatic clustering of biological data"

Jiarui Ding [1,2], Sohrab Shah [1,2] and Anne Condon [1]

[1]Department of Computer Science, University of British Columbia, Vancouver, BC V6T 1Z4, Canada

[2]Department of Molecular Oncology, BC Cancer Research Centre, 675 West 10th Avenue, Vancouver, BC V5Z 1L3, Canada

1 ALGORITHM

The densityCut algorithm shares some advantages of both density-based algorithms and spectral clustering algorithms and is summarized in Supplementary Algorithm 1. In the algorithm,

$$V_K(\mathbf{x}) = V_D \times (r_K(\mathbf{x}))^D$$

is the volume of the smallest ball centred at $\mathbf{x}$ containing K points from $\mathcal{D}$. V_D is the volume of the unit ball in the D-dimensional space, and $r_K(\mathbf{x})$ is the distance from $\mathbf{x}$ to its Kth nearest neighbour. Equation 9 and Equation 10 are from the main text.

2 COMPARING CLUSTERINGS

Three kinds of measures have been developed in the literature to assess the similarity between two clusterings (Meilă, 2007; Wagner *et al.*, 2007). The first type of measure is based on set overlaps, i.e., to match two clusterings such that the absolute or relative overlap is maximized. The second type of measure originates in information theory and is based on mutual information, i.e., our knowledge about one clustering increasing when we are told the other clustering. The third type of measure is based on counting pairs, i.e., to consider $\binom{N}{2}$ pair of decisions of assigning a point from clustering one and a point from clustering two to separate clusters or the same cluster. In this study, we used three measures, one from each type to compare clusterings. In addition, we can calculate these measures between a clustering and the ground truth (if available) to assess the accuracy of the clustering.

We first introduce the notations used in defining different measures. Let a clustering $\mathcal{C} = \{C_1, \ldots, C_L\}$ is a partition of the dataset $\mathcal{D} = \{\mathbf{x}^i\}_{i=1}^N$ into L mutually disjoint subsets $C_i, i \in 1, \ldots, L$. Let $\mathcal{C}' = \{C'_1, \ldots, C'_R\}$ denotes a second clustering of $\mathcal{D}$. Let element $M_{i,j} = |C_i \cap C'_j|$ denotes the $(i,j)^{th}$ entry of the confusion matrix $\mathbf{M}^{L \times R}$ between $\mathcal{C}$ and $\mathcal{C}'$. In other words, $M_{i,j}$ denotes the number of points that are common in cluster C_i and C'_j.

The maximum-matching measure (MMM) (Meilă, 2007; Wagner *et al.*, 2007) is based on set overlaps, and it is a generalization of the accuracy in classification applications. It defines a mapping between $\mathcal{C}$ and $\mathcal{C}'$, such that the sum of the number of common points between $\mathcal{C}$ and $\mathcal{C}'$ ($\bar{M}$) is maximized, under the constraint that only one entry in $\mathcal{C}$ ($\mathcal{C}'$) can match one entry in $\mathcal{C}'$ ($\mathcal{C}$). Then the

Supplementary Algorithm 1 The densityCut algorithm. Unless otherwise specified, all results in this paper are obtained with $K = \log_2(N)$ and $\alpha = 0.9$.

Input

- A set of data points $\mathcal{D} = \{\mathbf{x}_i\}_{i=1}^N, \mathbf{x}_i \in \mathbb{R}^D$
- The number of nearest neighbours K
- The damping factor α

1. Density estimation

$$f_i^0 = \frac{(K-1)/N}{V_K(\mathbf{x}_i)}$$

$$W_{i,j} = \begin{cases} 1 & \mathbf{x}_j \in K\text{nn}(\mathbf{x}_i) \\ 0 & \text{otherwise} \end{cases}$$

2. Density refinement

$$P_{i,j} = \frac{W_{i,j}}{\sum_j W_{i,j}}$$

Iterate until $||\mathbf{f}^{t+1} - \mathbf{f}^t|| \le \epsilon$ (default value: $\epsilon = 10^{-6}$)

$$\mathbf{f}^{t+1} = \alpha \mathbf{f}^t \mathbf{P} + (1-\alpha)\mathbf{f}^0$$

3. Local-maxima based clustering

Detect modes, i.e., local maxima, of the underling density function from

$$\{v_j \mid \forall P_{i,j} > 0, f_i < f_j\}$$

Build trees of points rooted at the modes, using

$$\text{Parent}(v_i) = \arg\min_{v_j \in \mathcal{N}_i} (|d_j - d_i| \mid f_i < f_j)$$

Build one cluster per tree, containing all points in that tree

4. Hierarchical stable clustering

Calculate heights of trees and valleys

(Optional) adjust valley heights based on Equation 10

Compute the saliency index ν for a pair of adjacent trees (Equation 9)

Merge clusters to generate a hierarchical tree by varying ν,

$\nu \in \{0.0, 0.05, 0.10, \ldots, 0.95, 1.0\}$

Select the most stable clustering

© Oxford University Press .

MMM between $\mathcal{C}$ and $\mathcal{C}'$ is defined as:

$$\text{MMM}(\mathcal{C}, \mathcal{C}') = \frac{\bar{M}}{N} \quad (1)$$

For perfect match $\text{MMM}(\mathcal{C}, \mathcal{C}') = 1$. However, unlike accuracy for classification, the maximum-matching measure between two random clusterings is not zero. In fact, the minimum maximum-matching measure is $1/N$ under the extrem condition that $L = N$ and $R = 1$.

The normalized mutual information (NMI) between $\mathcal{C}$ and $\mathcal{C}'$ is defined as follows (Ana *et al.*, 2003):

$$\text{NMI}(\mathcal{C}, \mathcal{C}') = \frac{2I(\mathcal{C}, \mathcal{C}')}{H(\mathcal{C}) + H(\mathcal{C}')} \quad (2)$$

where $H(\mathcal{C}) = -\sum_{i=1}^{L} \frac{|C_i|}{N} \log_2(\frac{|C_i|}{N})$ is the entropy associated with clustering $\mathcal{C}$. The mutual information between clustering $\mathcal{C}$ and $\mathcal{C}'$ is defined as $I(\mathcal{C}, \mathcal{C}') = \sum_i \sum_j \frac{|C_i \cap C_j|}{N} \log_2(\frac{|C_i \cap C_j|/N}{|C_i|/N * |C_j|/N})$. The normalized mutual information is a number between 0 and 1. For perfect match, $\text{NMI}(\mathcal{C}, \mathcal{C}') = 1$, and $\text{NMI}(\mathcal{C}, \mathcal{C}') = 0$ if the joint distribution $P_{i,j} = \frac{|C_i \cap C_j|}{N}$ is independent. Because of the strong independent requirement, the NMI between two random clusterings is typically a small number but not zero.

The adjusted Rand index (ARI) compares pair of assignments form $\mathcal{C}$ and $\mathcal{C}'$, and is defined as:

$$\text{ARI}(\mathcal{C}, \mathcal{C}') = \frac{t_0 - t_3}{(t_1 + t_2)/2 - t_3} \quad (3)$$

where $t_0 = \sum_{i=1}^{L} \sum_{j=1}^{R} \binom{M_{i,j}}{2}$, $t_1 = \sum_{i=1}^{k} \binom{|C_i|}{2}$, $t_2 = \sum_{j=1}^{l} \binom{|C'_j|}{2}$, and $t_3 = t_1 t_2 / \binom{N}{2}$. Compared to MMM and NMI, ARI has been corrected for chance, i.e., $\text{ARI}(\mathcal{C}, \mathcal{C}') = 0$ when the elements of the confusion matrix $\mathbf{M}$ follow a generalized geometric distribution (the two clusterings $\mathcal{C}$ and $\mathcal{C}'$ are picked at random, subjected to having the original number of elements in each cluster (Hubert *et al.*, 1985). In other words, the marginal distributions of the confusion matrix $\mathbf{M}$ are the same as the originals.) An undesired property of the adjusted Rand index is that negative values can occur. For perfect match, $\text{ARI}(\mathcal{C}, \mathcal{C}') = 1$.

3 COMPETING ALGORITHMS

We compared densityCut with three best algorithms reported in Wiwie *et al.* (2015), i.e., the hierarchical clustering algorithm (HC, from the R stats package) with average linkage, the partitioning around medoids (PAM, from the R cluster package) algorithm, and the density-based clustering algorithm OPTICS (Ankerst *et al.*, 1999) (from the R dbscan package). Notice that in Wiwie *et al.* (2015), two density-based algorithms (DBSCAN (Ester *et al.*, 1996) and clusterdp (Rodriguez *et al.*, 2014)) were tested and showed good performance. Currently the clusterdp algorithm needs some human interactions to select the cluster centers, and unfortunately there is no agreed way to automatically set this parameter. Similarly, DBSCAN is very sensitive to the parameter epsilon, which is the radius used to define the neighbours for each data point. We therefore used the OPTICS algorithm, which is similar to DBSCAN, but is more robust because essentially there is no need to set the epsilon parameters. We extracted clusters from OPTICS outputs based on the methods of Sander et al, 2003. The points considered as outliers by OPTICS were assigned to other clusters by a K-nearest neighbour classifier (where K is the same as the MinPts parameter of OPTICS).

We did not compare densityCut to one of the best clustering tools reported in Wiwie *et al.* (2015), transitivity clustering (Wittkop *et al.*, 2010), because we could not find an easy to use software package for clustering large datasets represented as matrices. We also compared densityCut with the Gaussian mixture model (GMM, implemented in the R mclust package (Fraley *et al.*, 2007)) based clustering algorithm and the normalized cut (NCut, implemented in the kernlab package (Zeileis *et al.*, 2004)) spectral clustering algorithm. These algorithms generally represent broad classes of methods for clustering analysis (i.e., hierarchical, partition, density-based, model-based, and graph-based) (Andreopoulos *et al.*, 2009)

4 SYNTHETIC DATASETS

We used ten synthetic datasets in our study (downloaded from `http://cs.joensuu.fi/sipu/datasets/`). Fig. 4 shows these synthetic datasets: Aggregation (Gionis *et al.*, 2007), Compound (Zahn, 1971), Flame (Fu *et al.*, 2007), Spiral (Chang *et al.*, 2008), Jain (Jain *et al.*, 2005), Pathbased (Chang *et al.*, 2008), R15 (Veenman *et al.*, 2002), D31 (Veenman *et al.*, 2002), S3 (Fränti and Virmajoki, 2006), and S4 (Fränti and Virmajoki, 2006). The number of data points in each dataset is 788, 399, 240, 312, 373, 300, 600, 3100, 5000, and 5000, respectively. Seven out of the ten datasets have been used in Wiwie *et al.* (2015) to compare various clustering algorithms (except for Jain, D31, and S4).

5 MICROARRAY GENE EXPRESSION DATA

The first microarray gene expression dataset consists of the expression of 1543 genes from four types of lung cancer tissues (186 snap-frozen tumours) and 17 normal lung tissues (Bhattacharjee *et al.*, 2001). These lung tumours include 139 adenocarcinomas, 21 squamous cell lung carcinomas, 20 pulmonary carcinoids, 6 small cell lung cancer. This dataset was downloaded from `http://bioinformatics.rutgers.edu/Static/Supplements/CompCancer/Affymetrix/bhattacharjee-2001/bhattacharjee-2001_database.txt`. The performance of different clustering algorithms on this dataset is in Supplementary Fig. 6.

The second microarray gene expression dataset consists of the expression of 182 genes from the mixture of breast cancer tissues and colon cancer tissues (Chowdary *et al.*, 2006). The breast tumours consist of 32 pairs of snap-frozen tumours and the corresponding preserved tumours, and the colon tumours consist of 20 pairs of snap-frozen tumours and the corresponding preserved tumours. This dataset was downloaded from `http://bioinformatics.rutgers.edu/Static/Supplements/CompCancer/Affymetrix/chowdary-2006/chowdary-2006_database.txt`.

REFERENCES

Ana, L. *et al.* (2003). Robust data clustering. In *CVPR*, volume 2, pages 128–133.

Andreopoulos, B. *et al.* (2009). A roadmap of clustering algorithms: finding a match for a biomedical application. *Brief. Bioinform.*, **10**(3), 297–314.

Ankerst, M. *et al.* (1999). OPTICS: ordering points to identify the clustering structure. In *SIGMOD Rec.*, volume 28, pages 49–60. ACM.

Bhattacharjee, A. *et al.* (2001). Classification of human lung carcinomas by mrna expression profiling reveals distinct adenocarcinoma subclasses. *Proc. Natl. Acad. Sci. USA*, **98**(24), 13790–13795.

Chang, H. *et al.* (2008). Robust path-based spectral clustering. *Pattern Recog.*, **41**(1), 191–203.

Chowdary, D. *et al.* (2006). Prognostic gene expression signatures can be measured in tissues collected in rnalater preservative. *J. Mol. Diagn.*, **8**(1), 31–39.

Ester, M. *et al.* (1996). A density-based algorithm for discovering clusters in large spatial databases with noise. In *KDD*, pages 226–231.

Fraley, C. *et al.* (2007). Model-based methods of classification: using the mclust software in chemometrics. *J. Stat. Softw.*, **18**(6), 1–13.

Fränti, P. and Virmajoki, O. (2006). Iterative shrinking method for clustering problems. *Pattern Recog.*, **39**(5), 761–775.

Fu, L. *et al.* (2007). FLAME, a novel fuzzy clustering method for the analysis of dna microarray data. *BMC Bioinform.*, **8**(1).

Gionis, A. *et al.* (2007). Clustering aggregation. *ACM Trans. Knowl. Discov. Data*, **1**(1).

Hubert, L. *et al.* (1985). Comparing partitions. *J. classif.*, **2**(1), 193–218.

Jain, A. K. *et al.* (2005). Data clustering: a user's dilemma. In *Pattern Recog. Mach. Intell.*, volume 3776 of *LNCS*, pages 1–10. Springer-Verlag.

Meilă, M. (2007). Comparing clusterings–an information based distance. *J. Multivar. Anal.*, **98**(5), 873–895.

Rodriguez, A. *et al.* (2014). Clustering by fast search and find of density peaks. *Science*, **344**(6191), 1492–1496.

Veenman, C. J. *et al.* (2002). A maximum variance cluster algorithm. *IEEE Trans. Pattern Anal. Mach. Intell.*, **24**(9), 1273–1280.

Wagner, S. *et al.* (2007). Comparing clusterings – an overview. Technical Report 2006-04, Universität Karlsruhe.

Wittkop, T. *et al.* (2010). Partitioning biological data with transitivity clustering. *Nat. Methods*, **7**(6), 419–420.

Wiwie, C. *et al.* (2015). Comparing the performance of biomedical clustering methods. *Nature methods*, **12**(11), 1033–1038.

Zahn, C. T. (1971). Graph-theoretical methods for detecting and describing gestalt clusters. *IEEE Trans. Comput.*, **100**(1), 68–86.

Zeileis, A. *et al.* (2004). kernlab-an S4 package for kernel methods in R. *J. Stat Softw.*, **11**(9), 1–20.

SUPPLEMENTARY FIGURES

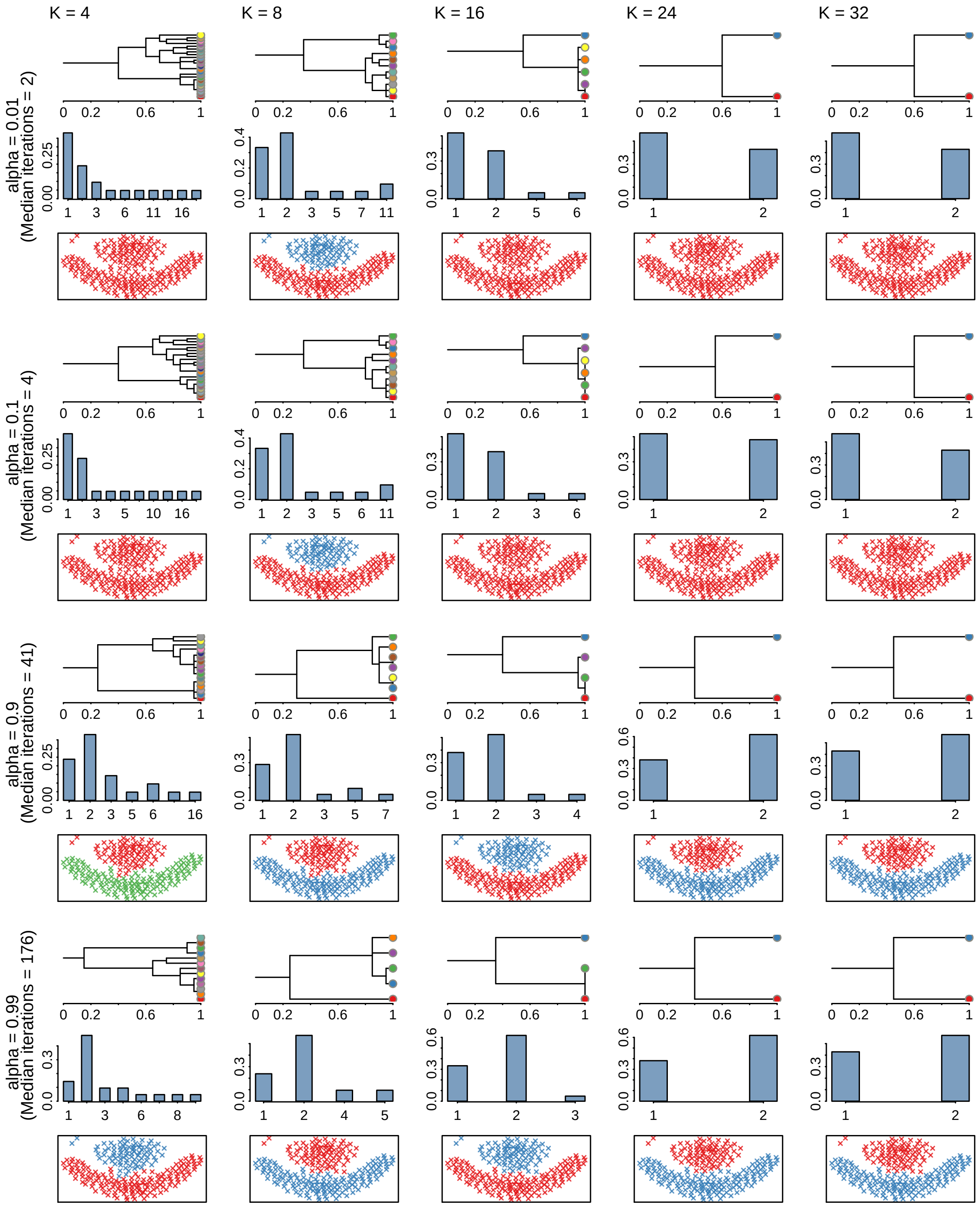

Supplementary Fig. 1: The influence of densityCut parameter K and α on the final clustering results. When $K = \log_2(N) = 8$, `densityCut` correctly detected the two clusters given different values for α. Small $K = \log_2(N) = 4$ produced 'spiky' density estimates and resulted in many local maxima. Large K produced flat density estimates, and the two true clusters tended to merge because of no deep valley between them. In addition, when $\alpha = 0.9$ or 0.99, `densityCut` correctly detected the two clusters given different values for K. Increasing α produced better clustering results but it took much longer for the density refinement step to converge, e.g., median 176 iterations when $\alpha = 0.99$ compared to 41 iterations when $\alpha = 0.90$.

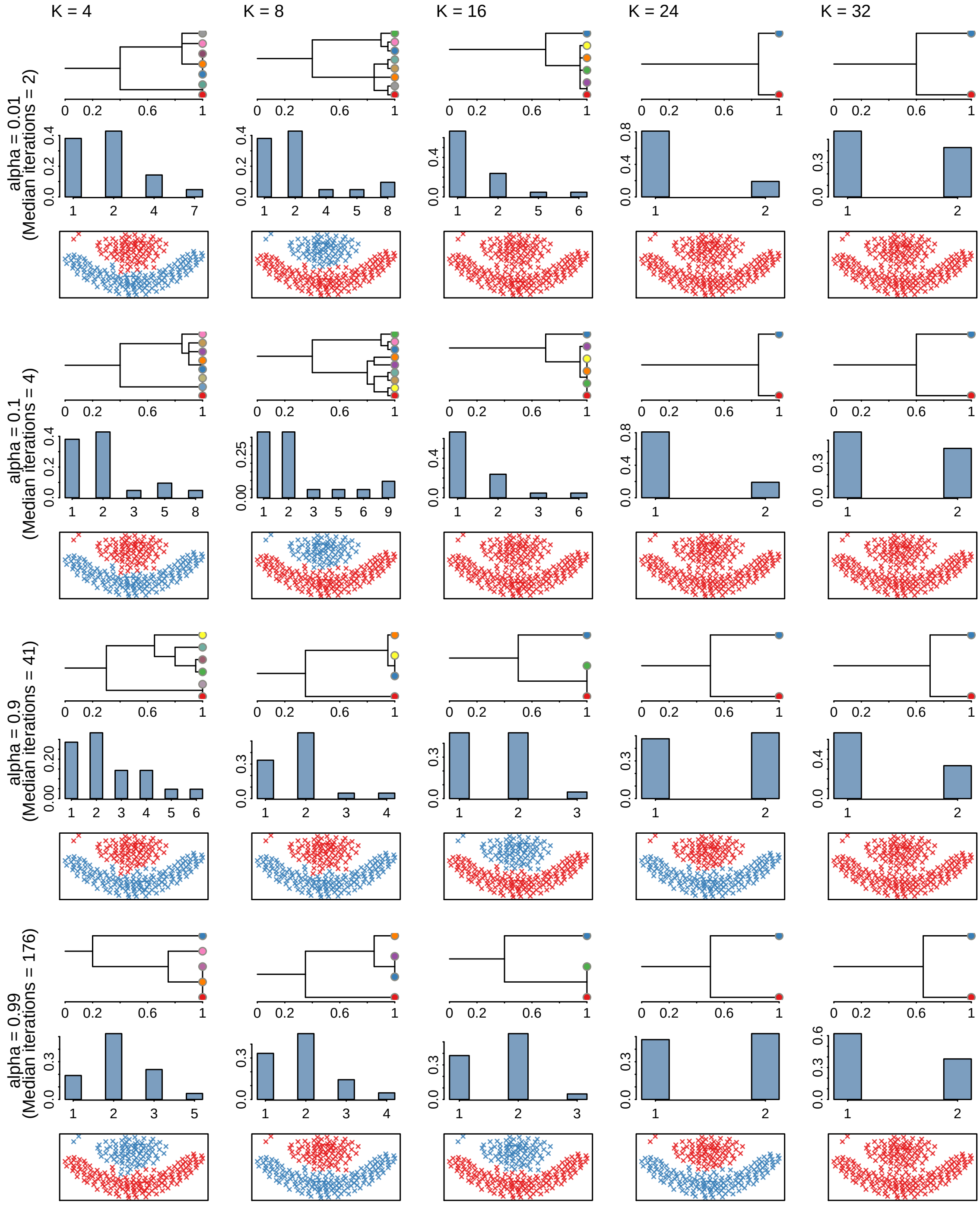

Supplementary Fig. 2: The influence of `densityCut` parameter K and α on the final clustering results (with the valley height adjustment step). The valley height adjustment step plays a role of smoothing the density estimates. This functionality is especially useful for small K. For example, when $K = 0.5 \log 2(N) = 4$, densityCut correctly detected the two clusters after adjusting the heights of valleys.

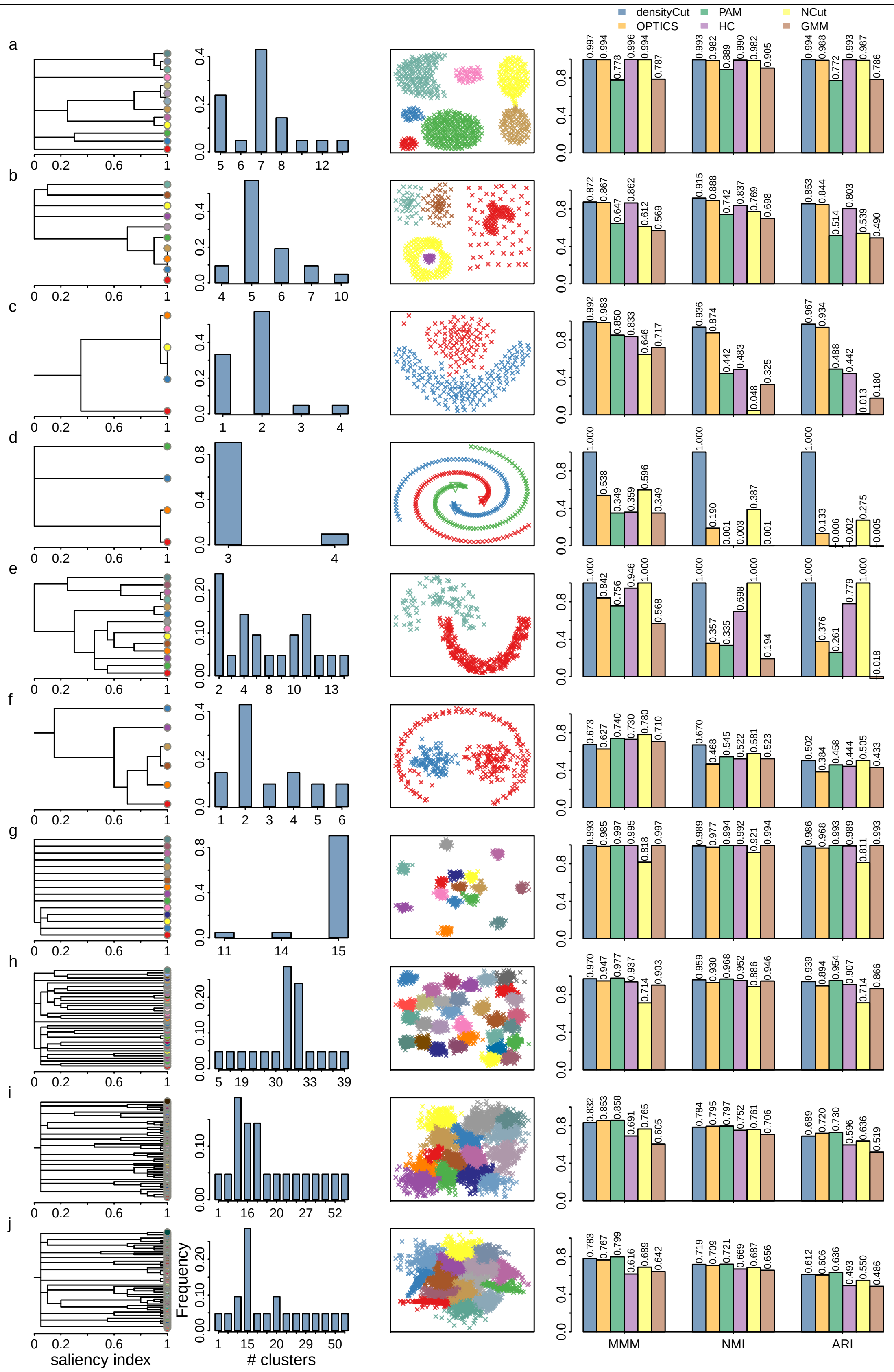

Supplementary Fig. 3: Results on the synthetic benchmark datasets consisting of irregular, non-convex or overlapped clusters. First-column figures show the clustering trees, the second-column figures show the cluster number frequency plots, the third-column figures show the final clustering results, and the fourth-column figures show the maximum-matching measure (MMM), the normalized mutual information (NMI), and the adjusted Rand index (ARI) from comparing clustering results of each algorithm to the ground truth.

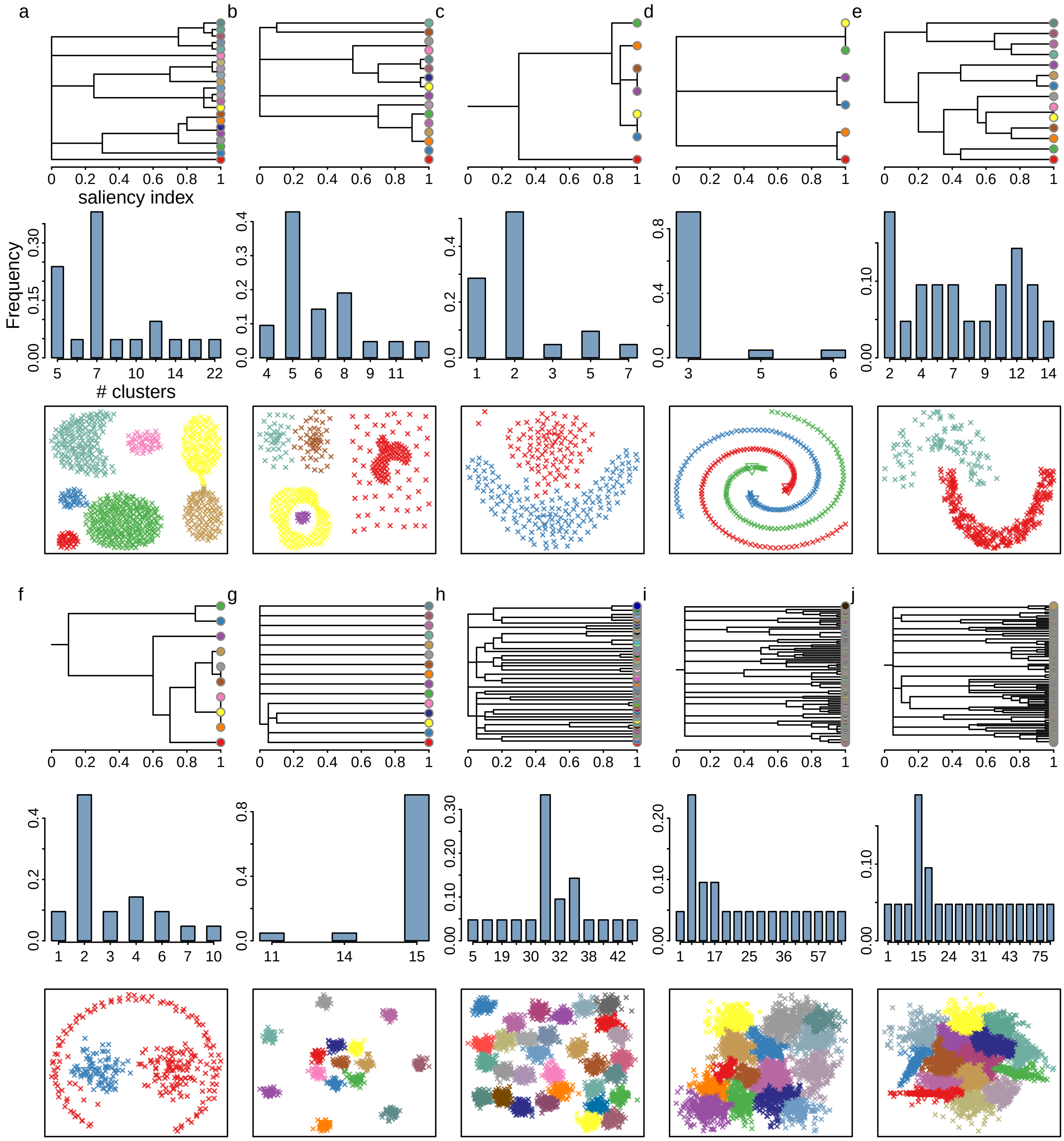

Supplementary Fig. 4: `densityCut` results on the synthetic benchmark datasets without the valley height adjustment step. The results are exactly the same as those obtained with the valley height adjustment step.

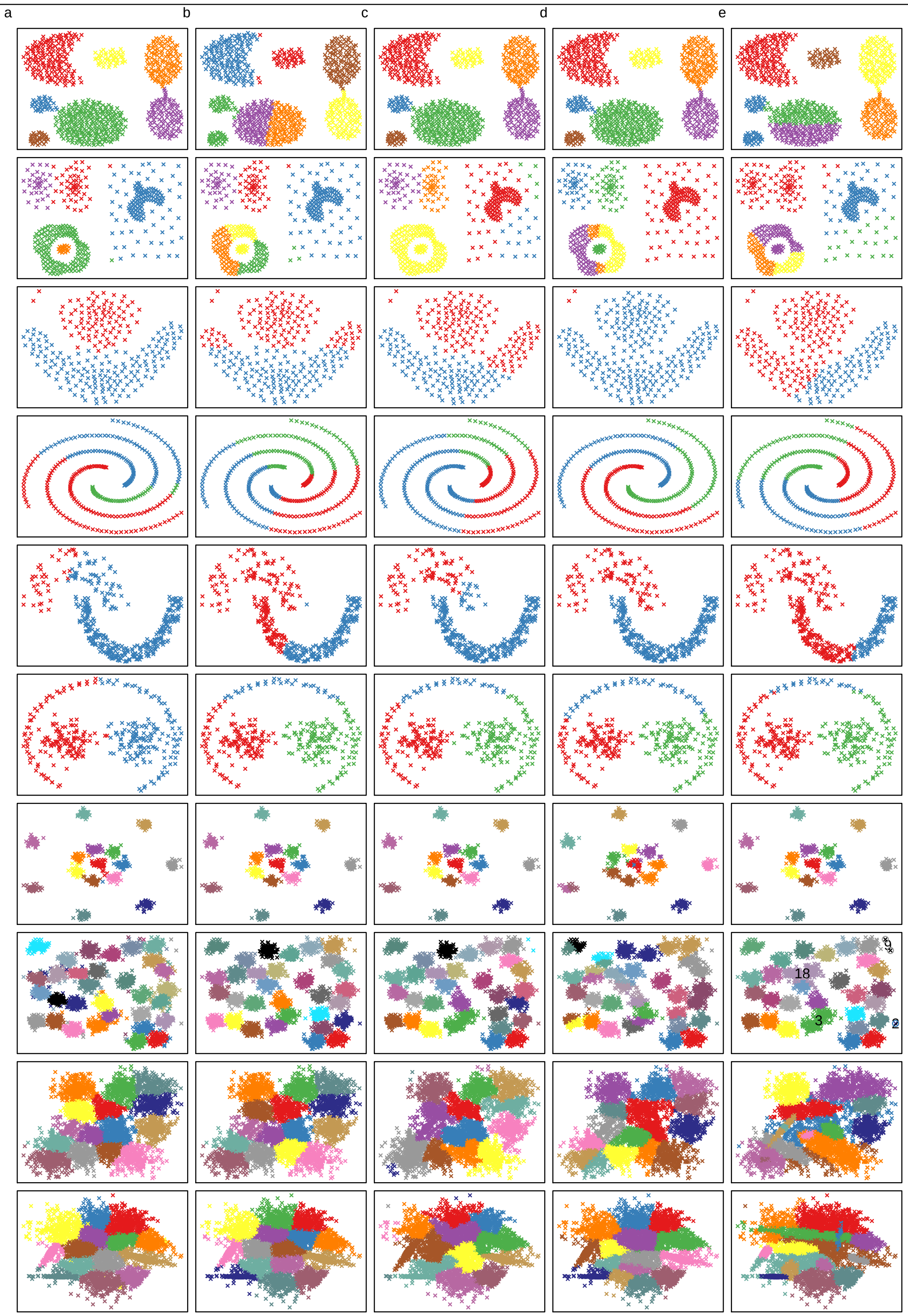

Supplementary Fig. 5: Scatter plots show the results of different cluster algorithms on the synthetic benchmark datasets consisting of irregular, un-convex shape or overlapping clusters. (a) Density based clustering OPTICS, (b) PAM, (c) hierarchical clustering with average linkage HC, (d) normalized cut spectral cluster algorithm NCut, (e) Gaussian mixture model based clustering algorithm GMM.

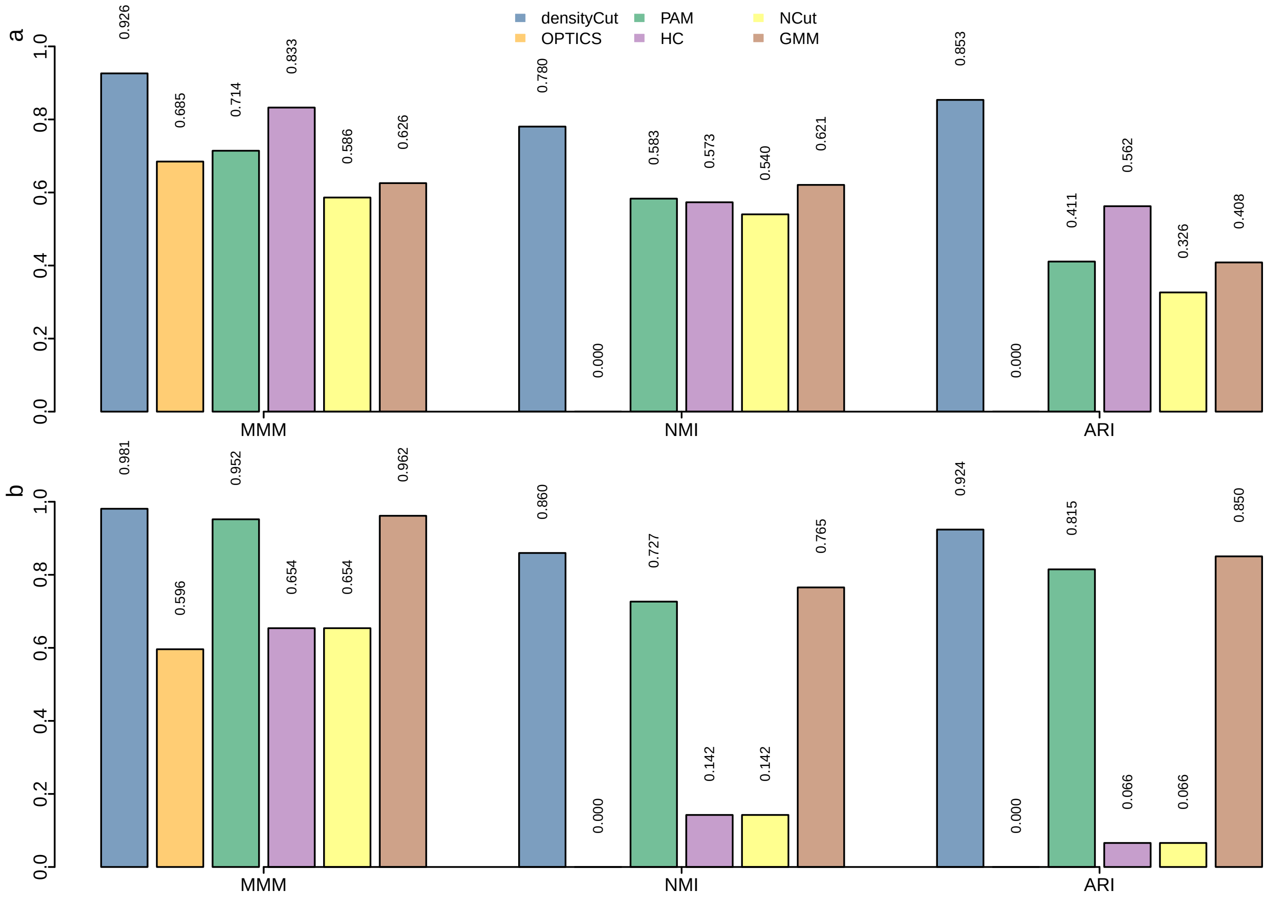

Supplementary Fig. 6: Clustering microarray gene expression data. Clustering results on (a) the lung cancer dataset, and (b) the mixture of breast cancer and colon cancer dataset.

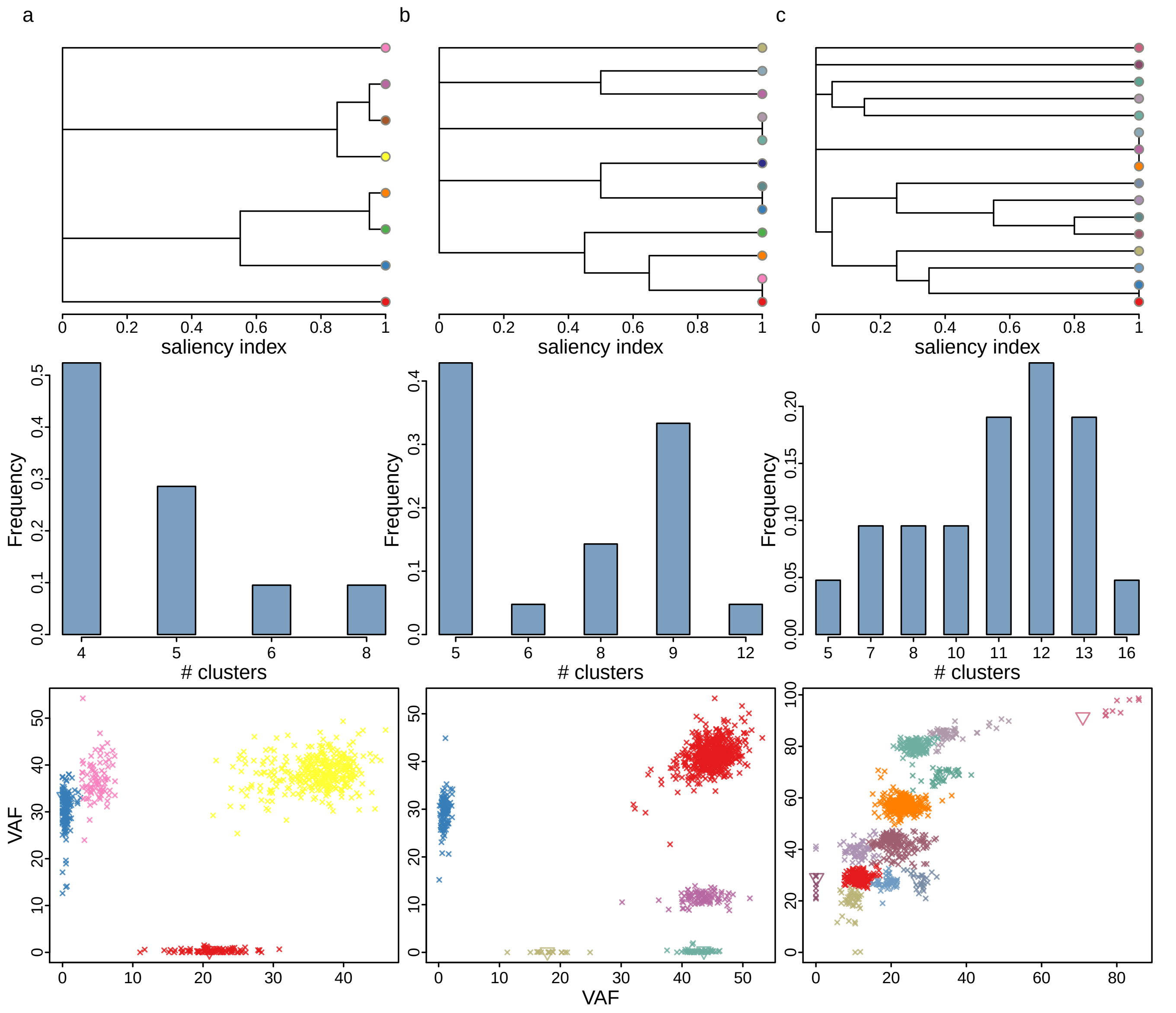

Supplementary Fig. 7: Clustering variant allele frequencies of somatic mutations using `densityCut`. (a) Clustering multi-time sample data from initial primary myelofibrosis (PMF), acute myeloid leukaemia (AML), and after treatment relapsed PMF using `densityCut`. (b) Clustering the somatic mutations from sequencing a primary/relapse pair of an AML patient using `densityCut`. (c) Clustering the somatic mutations from sequencing a lung/pancreas metastasis pair of an melanoma patient using `densityCut`. First row figures show the clustering trees, the second row figures show the cluster number frequency plots, and the third row figures show the final clustering results. We used the default parameter setting for clustering analysis.

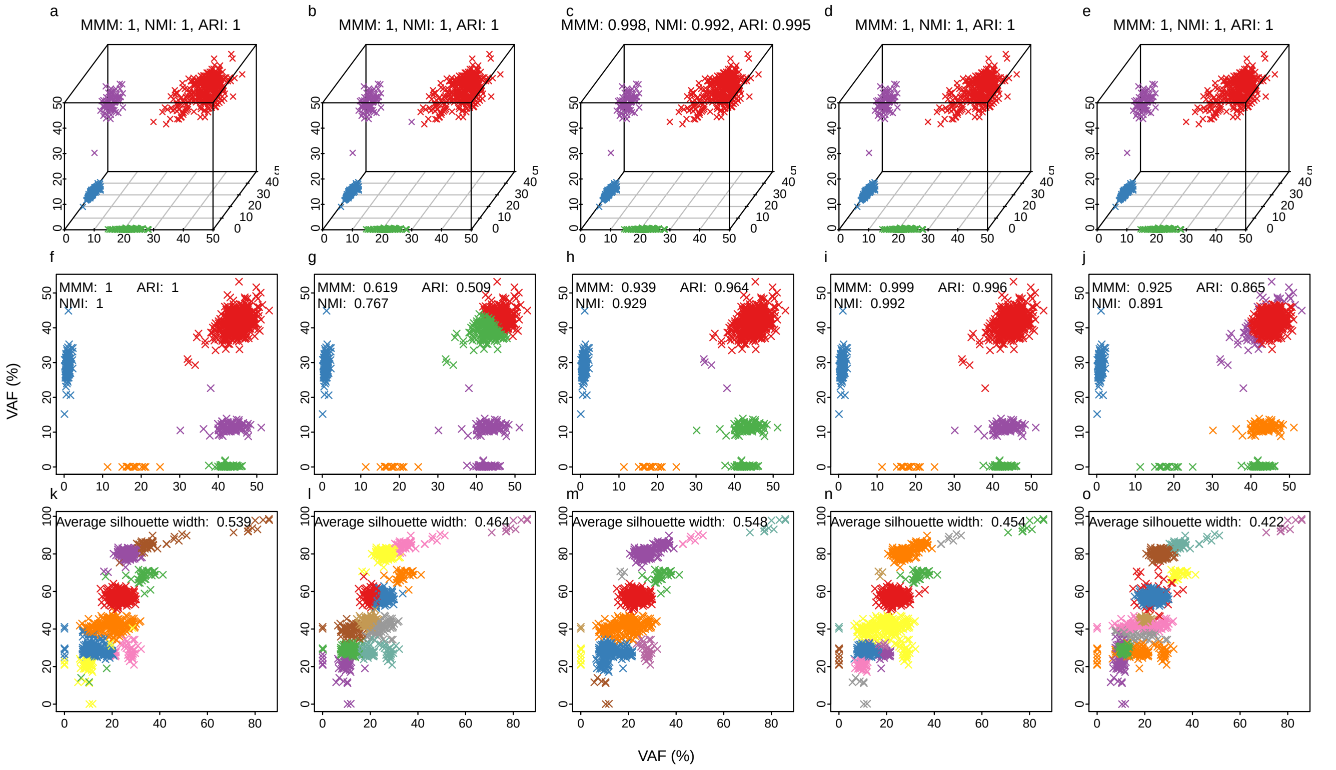

Supplementary Fig. 8: Clustering variant allele frequencies of somatic mutations. (a-e) Clustering multi-time sample data from initial primary myelofibrosis (PMF), acute myeloid leukaemia (AML), and after treatment relapsed PMF. (f-j) Clustering the somatic mutations from sequencing a primary/relapse pair of an AML patient. (k-o) Clustering the somatic mutations from sequencing a lung/pancreas metastasis pair of an melanoma patient. First column figures show the OPTICS clustering results, the second column figures show the PAM clustering results, the third column figures show the HC clustering results, the fourth column figures show the NCut clustering results, and the fifth column figures show the GMM clustering results,.

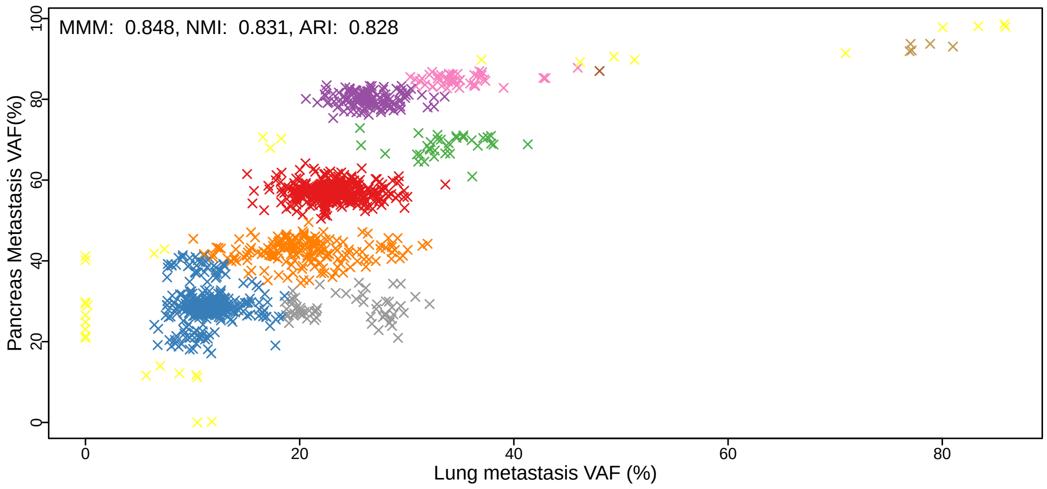

Supplementary Fig. 9: Clustering the somatic mutations from sequencing a lung/pancreas metastasis pair of a melanoma patient using sciClone (without considering copy number alterations). The yellow colour cluster may be meaningful in terms of clustering because it models the ‘outliers’. However, it may not have biological meaning because the mutations in this cluster could from different clones. The MMM, NMI and ARI were computed from comparing sciClone results (ten clusters) with the `densityCut` results (12 clusters).

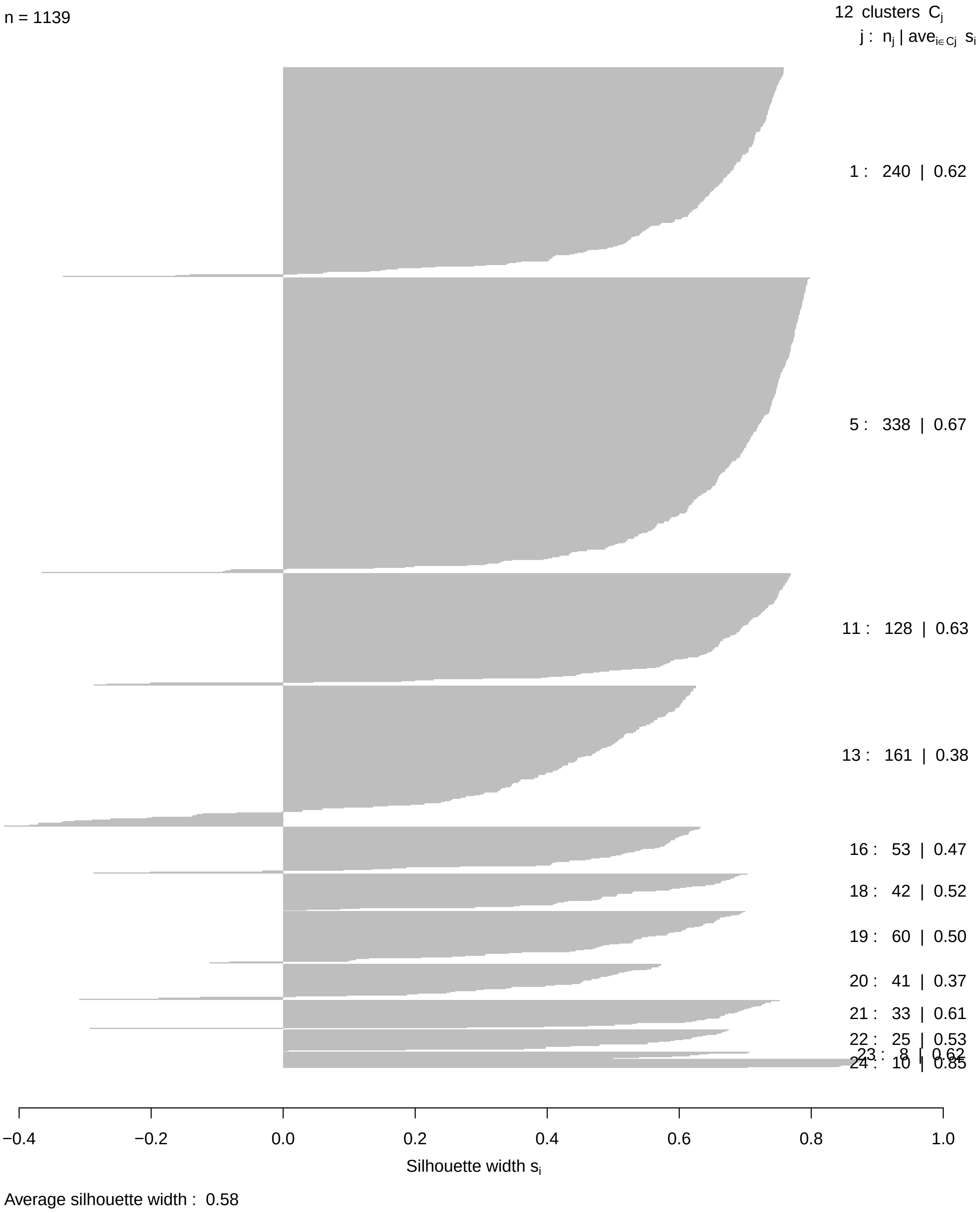

Supplementary Fig. 10: Plot of silhouette values from clustering of the variant allele frequencies of somatic mutations from sequencing a melanoma lung/pancreas metastasis pair by `dencityCut`. The high average silhouette width of 0.58 suggests that this dataset could contain 12 clusters.

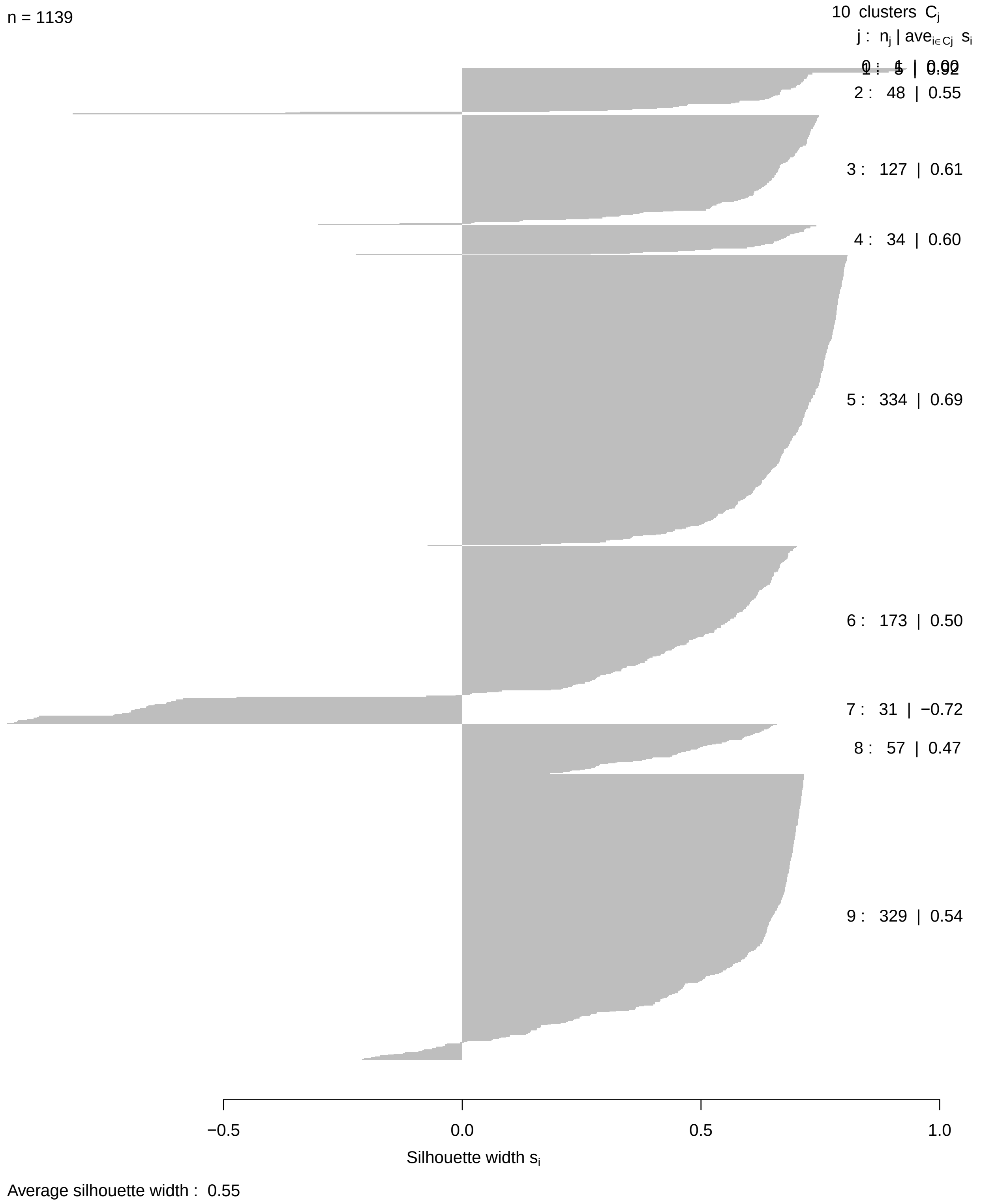

Supplementary Fig. 11: Plot of silhouette values from clustering of the variant allele frequencies of somatic mutations from sequencing a melanoma lung/pancreas metastasis pair by sciClone.

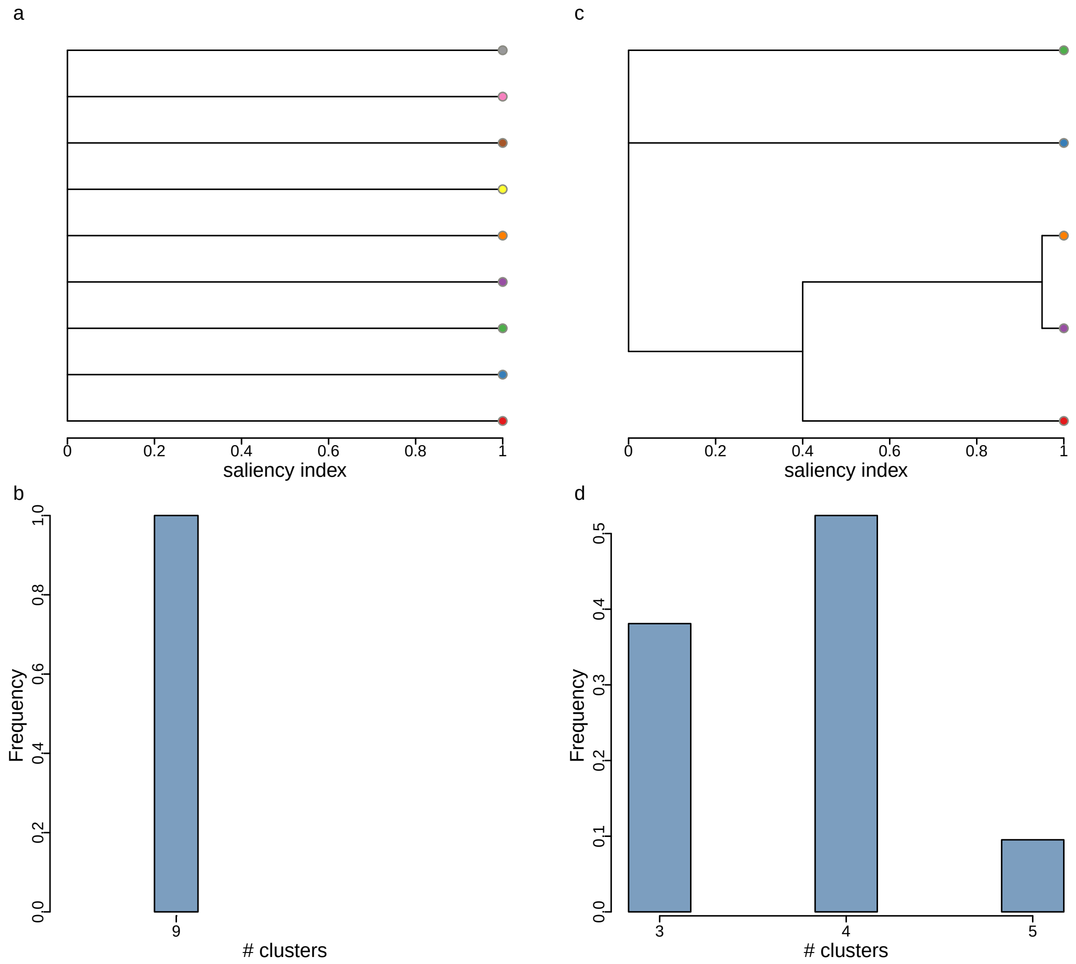

Supplementary Fig. 12: Clustering single-cell RNA-seq data. (a-b) Clustering the gene expression data of 301 cells. `densityCut` detected nine distinct clusters. (c-d) Clustering the expression data of 223 stem cells from the sub ventricular zone of eight-week-old mice. The most stable clustering is four clusters. First row figures show the clustering trees, and the second row figures show the cluster number frequency plots.

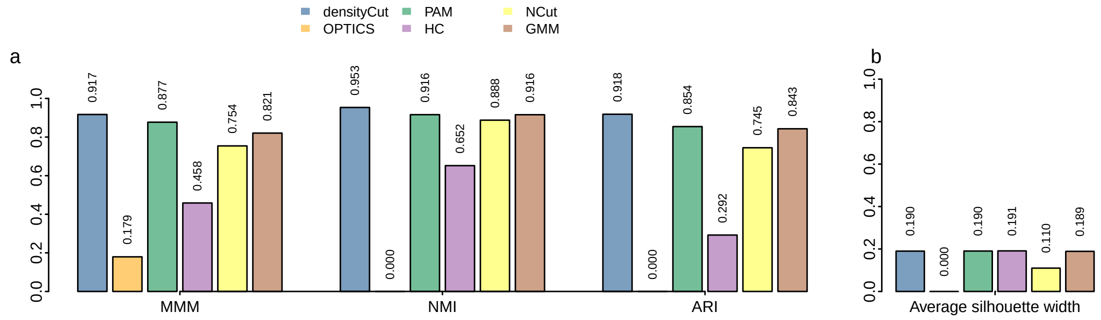

Supplementary Fig. 13: Performance measures on clustering single-cell gene expression data. (a) The clustering measures from clustering the gene expression data of 301 cells. (b) Average silhouette widths from clustering the expression data of 223 stem cells from the sub ventricular zone of eight-week-old mice.

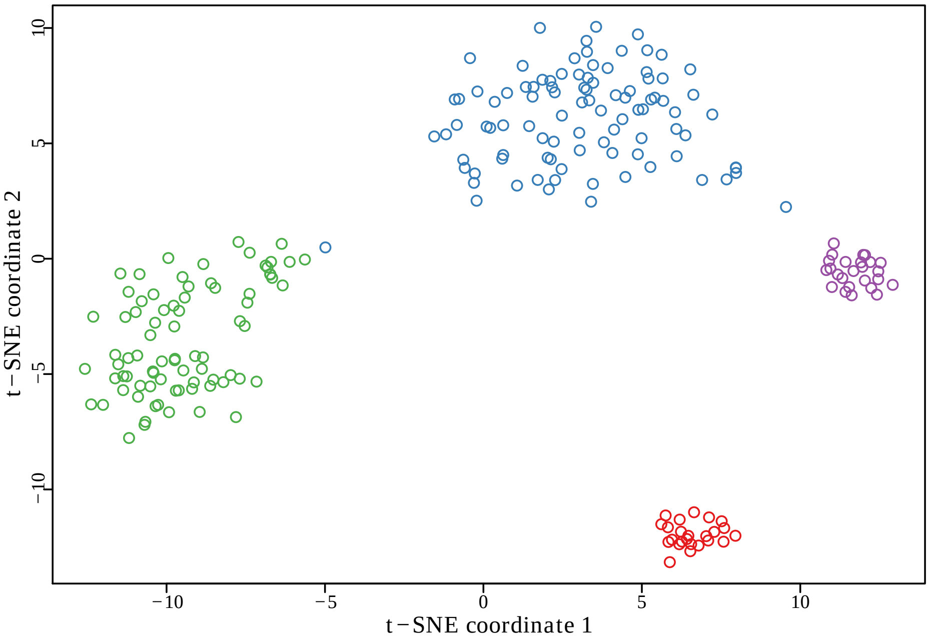

Supplementary Fig. 14: Visualizing the mouse brain stem cell expression data by t-Distributed Stochastic Neighbour Embedding (t-SNE). We can see four distinct clusters.

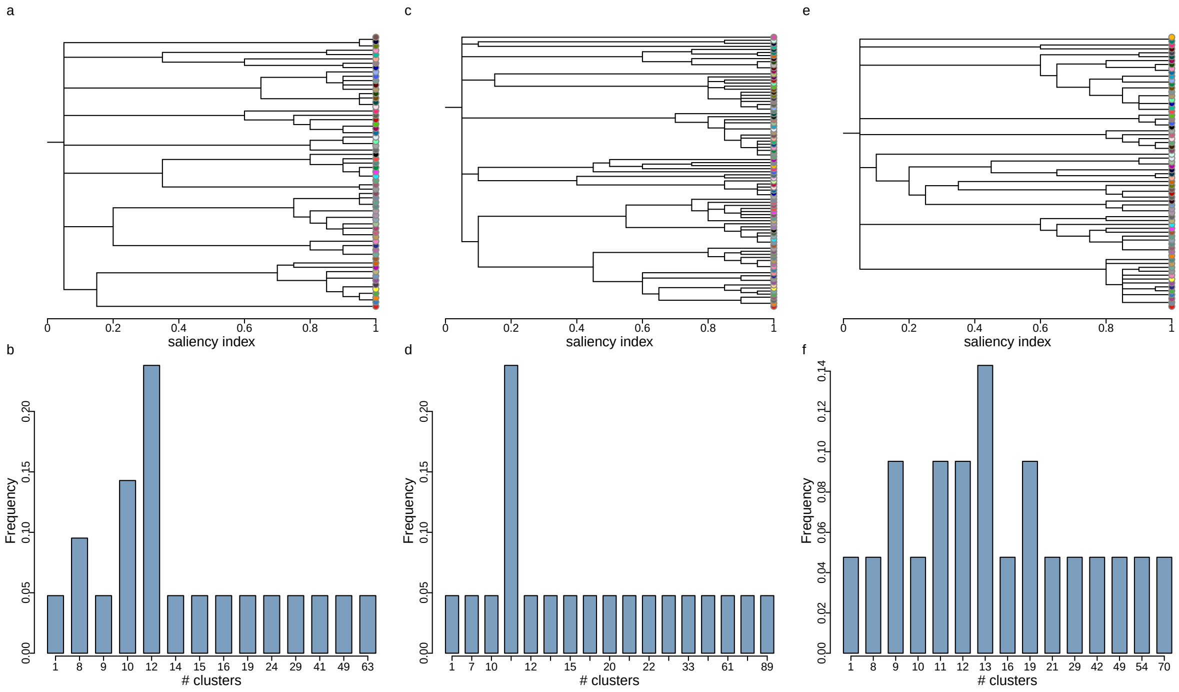

Supplementary Fig. 15: Clustering single-cell mass cytometry data. (a-b) Clustering benchmark CyTOF dataset one consisting of 81,747 cells. (c-d) Clustering benchmark CyTOF dataset two from heathy donor H1 consisting of 72473 cells. (e-f) Clustering benchmark CyTOF dataset two from heathy donor H2 consisting of 31,721 cells. First row figures show the clustering trees, and the second row figures show the cluster number frequency plots.

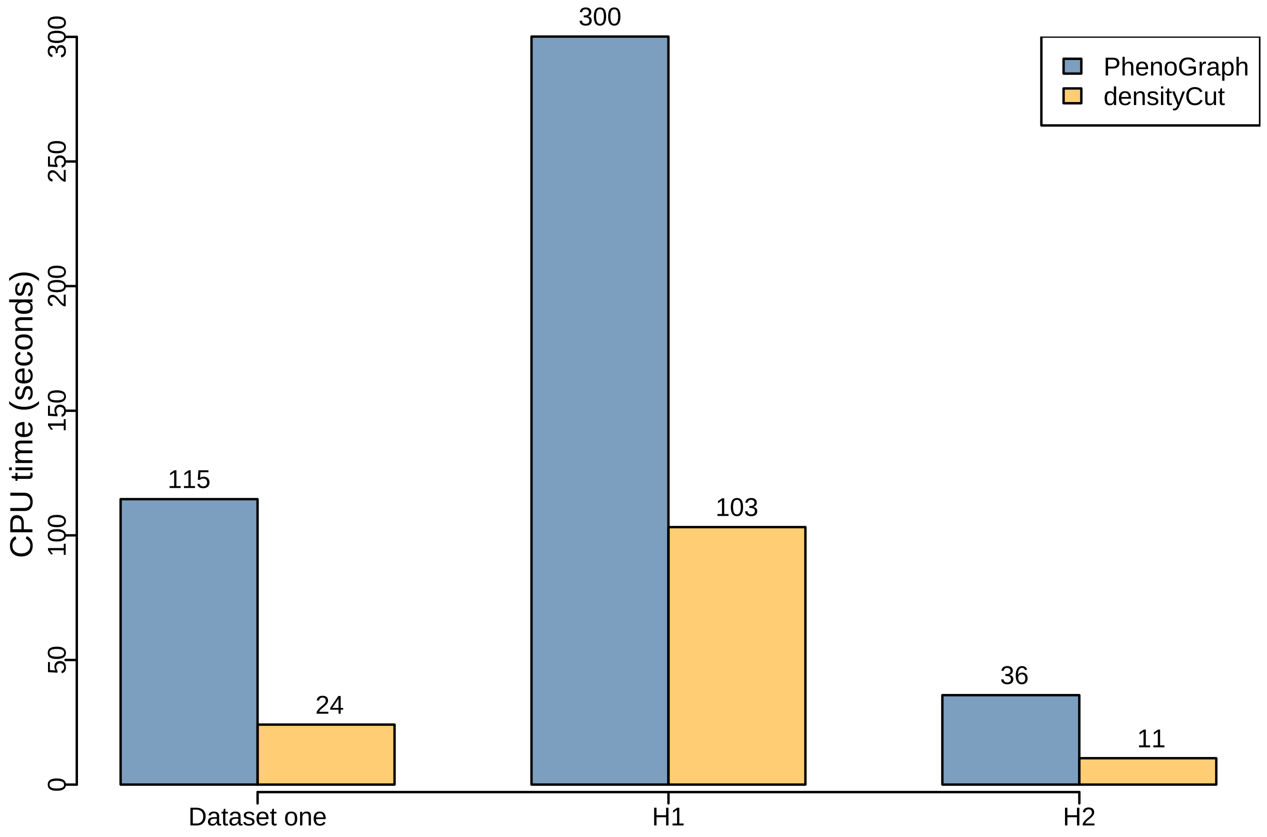

Supplementary Fig. 16: Comparing the time used by PhenoGraph and `densityCut` in clustering the benchmark CyTOF datasets. Although directly comparison is difficult since PhenoGraph is implemented in Python and `densityCut` is implemented in R, `densityCut` is around two times faster than PhenoGraph based on the current implementations.
